# *Circ_0053943* complexed with IGF2BP3 drives uveal melanoma progression via regulating N6-methyladenosine modification of *Epidermal growth factor receptor*

**DOI:** 10.32604/or.2024.045972

**Published:** 2024-04-23

**Authors:** ANDI ZHAO, YUE WANG, ZIJIN WANG, QING SHAO, QI GONG, HUI ZHU, SHIYA SHEN, HU LIU, XUEJUAN CHEN

**Affiliations:** 1 Department of Ophthalmology, The First Affiliated Hospital with Nanjing Medical University, Nanjing, 210029, China; 2 The First Clinical Medical College, Nanjing Medical University, Nanjing, 211166, China; 3 Department of Ophthalmology, Nanjing First Hospital, Nanjing Medical University, Nanjing, 210006, China

**Keywords:** Uveal melanoma, *Hsa_circ_0053943*, IGF2BP3, *EGFR*, MAPK/ERK signaling pathway

## Abstract

Numerous studies have characterized the critical role of circular RNAs (circRNAs) as regulatory factors in the progression of multiple cancers. However, the biological functions of circRNAs and their underlying molecular mechanisms in the progression of uveal melanoma (UM) remain enigmatic. In this study, we identified a novel circRNA, *circ_0053943*, through re-analysis of UM microarray data and quantitative RT-PCR. *Circ_0053943* was found to be upregulated in UM and to promote the proliferation and metastatic ability of UM cells in both *in vitro* and *in vivo* settings. Mechanistically, *circ_0053943* was observed to bind to the KH1 and KH2 domains of insulin-like growth factor 2 mRNA-binding protein 3 (IGF2BP3), thereby enhancing the function of IGF2BP3 by stabilizing its target mRNA. RNA sequencing assays identified *epidermal growth factor receptor* (*EGFR*) as a target gene of *circ_0053943* and IGF2BP3 at the transcriptional level. Rescue assays demonstrated that *circ_0053943* exerts its biological function by stabilizing *EGFR* mRNA and regulating the downstream mitogen-activated protein kinase/extracellular signal-regulated kinase (MAPK/ERK) signaling pathway. Collectively, *circ_0053943* may promote UM progression by stabilizing *EGFR* mRNA and activating the MAPK/ERK signaling pathway through the formation of a *circ_0053943*/IGF2BP3/*EGFR* RNA-protein ternary complex, thus providing a potential biomarker and therapeutic target for UM.

## Introduction

Uveal melanoma (UM) stands out as the most prevalent primary intraocular malignant tumor in adults, originating from melanocytes of the uveal tract, which includes the iris, ciliary body, and choroid [1]. Owing to its ocular location, UM presents unique challenges for diagnosis, often complicating direct visualization. Consequently, clinicians rely on a combination of clinical examinations and advanced imaging modalities to confirm the presence of the tumor and characterize its properties, including ultrasound (US) echography and the standardized A-scan technique [2,3]. Despite aggressive management of the primary tumor, approximately 50% of patients eventually develop distant metastasis, particularly in the liver, leading to a grim prognosis within 5 months [4,5]. Given this bleak long-term clinical outlook and the limitations of current treatments, there exists an unmet medical need to investigate the mechanisms of UM tumorigenesis and assess novel effective therapies.

Several studies have identified an abundance of genetic aberrancies in UM, with mutations in *BAP1*, *SF3B1*, and *EIF1AX* playing essential roles in progression and metastasis, while activating mutations in *Gα11/Q* are closely related to UM oncogenesis in most cases [6,7]. Nevertheless, the precise molecular mechanisms underlying UM remain primarily unclear. Circular RNAs (circRNAs), a recently discovered subclass of noncoding RNAs characterized by higher resistance to RNase R due to their closed continuous loop structures, exhibit increased stability, making them more suitable for diagnostic biomarkers and therapeutic targets compared to other RNA types [8]. Indeed, mounting evidence suggests that circRNAs are involved in the metastasis and proliferation of various cancers by acting as microRNA (miRNA) sponges, interacting with RNA-binding proteins (RBPs), splicing pre-mRNA transcripts, and even encoding small peptides or proteins [9–11]. However, the number of published studies investigating circRNAs in UM is limited compared to other cancers [12,13]. Recently, microarray analysis identified several differentially expressed circRNAs between UM and normal uveal tissues [12]. Subsequently, another study verified and demonstrated that the upregulated circ_0119872, derived from the host gene *RAS guanyl-releasing protein 3* (*RasGRP3*), could promote UM tumorigenesis via sponging miRNA and serve as an important prognostic biomarker [13]. Therefore, to gain a better understanding of UM development and progression, we aim to explore novel insights into the role and mechanism of circRNAs in UM. Considering that upregulated circRNAs can be potential therapeutic targets, we have selected *circ_0053943*, also derived from the host gene *RasGRP3* and listed among the top 10 overregulated circRNAs [12], for further in-depth investigation.

N6-methyladenosine (m^6^A) represents the most abundant epitranscriptomic modification found in messenger RNAs (mRNAs), contributing to the tumorigenesis of multiple cancers [14–16]. This modification is governed by a methyltransferase complex (“writers”), demethylases (“erasers”), and RNA-binding proteins (“readers”). Insulin-like growth factor 2 mRNA-binding proteins (IGF2BPs, including IGF2BP1/2/3) are newly identified distinct m^6^A readers, facilitating the stability and translation of m^6^A-modified transcripts [17,18]. Previous studies have demonstrated that non-coding RNAs (ncRNAs) might participate in IGF2BPs-mediated functions and modulate the expression of target transcripts [19–21]. However, the involvement of IGF2BPs in UM tumorigenesis remains to be elucidated concerning how underlying ncRNAs mediate these functional biological processes.

In this study, we identified a novel circRNA (*hsa_circ_0053943*) serving as an RNA m^6^A reader-cooperator, assisting the m^6^A reader (IGF2BP3) in guarding m^6^A-modified *epidermal growth factor receptor* (*EGFR*) against decay and promoting tumorigenesis and metastasis in UM. Our findings suggest that *circ_0053943* may cooperate with IGF2BP3 in the post-transcriptional regulation of *EGFR*, highlighting the functional importance of the *circ_0053943*/IGF2BP3/*EGFR* mRNA-protein ternary complex in the invasion and metastasis of UM.

## Materials and Methods

### Human tissue specimens

A total of five UM tissues and five human normal melanocyte tissues were acquired from The First Affiliated Hospital of Nanjing Medical University. Two experienced histopathologists independently assessed the histological features of all specimens. The clinicopathological characteristics can be found in Suppl. Table 1. The study involving patients received approval from the Human Ethics Committee of The First Affiliated Hospital of Nanjing Medical University under the number 2022-SRFA-334, adhering to the Declaration of Helsinki. Written informed consent was obtained from each patient. Subsequently, all specimens were promptly frozen in liquid nitrogen and stored at −80°C until utilized.

### Cell culture

Following a prior investigation [13], six ocular melanoma cell lines (MUM2B [RRID: CVCL_3447], C918 [RRID: CVCL_8471], Mel 270 [RRID: CVCL_C302], Omm2.5 [RRID: CVCL_C307], OCM-1 [RRID: CVCL_6934], and OCM-1A [RRID: CVCL_6935]) and the human retinal pigment epithelial cell line (ARPE-19 [RRID: CVCL_0145]) were selected and procured from the Cell Bank of Type Culture Collection of the Chinese Academy of Sciences (Shanghai, China). Human primary umbilical vein endothelial cells (HUVECs [RRID: CVCL_2959]) were obtained from the American Type Culture Collection (ATCC, Manassas, VA, USA). Before the commencement of the study, all cell lines underwent confirmation using a short tandem repeat method, ensuring they were free of mycoplasma contamination. According to the manufacturers’ guidelines, all cell lines were cultured in recommended media and incubated at 37°C in a humidified incubator with 5% CO_2_.

### RNA extraction and quantitative real-time polymerase chain reaction (qRT–PCR)

The total RNA of cell lines was extracted using TRIzol reagent (Invitrogen, Waltham, MA, USA) and subsequently reverse transcribed into complementary DNA (cDNA) using the PrimeScript RT Reagent Kit (Takara, Osaka, Japan), following the manufacturer’s instructions. The subsequent qRT-PCR, using the SYBR Green Kit (Takara), was conducted on the StepOnePlus Real-Time PCR System (Applied Biosystems, Foster City, CA, USA). The ^−ΔΔ^C_T_ method was employed to assess the transcript levels of mRNA, with either *glyceraldehyde 3-phosphate dehydrogenase* (*GAPDH*) or *U6* serving as an internal control. The primer sequences used can be found in Suppl. Table 2.

### Transfection

RiboBio (Guangzhou, China) synthesized recombinant lentivirus containing short hairpin RNA targeting *circ_0053943* (sh-*circ_0053943*#1/2/3) and IGF2BP3 (sh-IGF2BP3#1/2), along with the full-length targeting sequence of *circ_0053943* and IGF2BP3, including the corresponding negative control. Obio (Shanghai, China) synthesized the IGF2BP3 full-length (FL) and truncation-mutation plasmids with a C-terminus 3× Flag tag. Small interfering RNA (siRNA) oligonucleotides targeting methyltransferase-like 3 (METTL3) and methyltransferase-like 14 (METTL14) were also synthesized by RiboBio. Transfection of short hairpin (shRNAs) and plasmid vectors was carried out using Lipofectamine 3000 (Invitrogen). The transfection efficiency was subsequently confirmed through qRT-PCR. Detailed sequences of shRNAs and siRNAs (for silencing) can be found in Suppl. Table 3.

### Cell proliferation assay and flow cytometry assay

Cell proliferation/growth was assessed using a Cell Counting Kit-8 (CCK-8; Dojindo Laboratories, Dojindo, Japan) and the 5-ethynyl-2-deoxyuridine (EDU) assay with the Cell-Light EdU Apollo 488 *In Vitro* Kit (RiboBio), following the manufacturer’s protocols.

For the cell cycle assay, treated cells were fixed in 75% alcohol overnight at −20°C. After three washes, fixed cells were stained with propidium iodide (PI) buffer using a Cell Cycle Analysis Kit (Beyotime, Shanghai, China). Cells were incubated with H_2_O_2_ (1 mM) for 4 h to stimulate apoptosis for cell apoptosis assays. Subsequently, cells were stained with Annexin VAPC/7-AAD Apoptosis Detection Kit (KeyGEN, Jiangsu, China) following the manufacturer’s protocol. Cell cycle distribution percentage and apoptotic rates were determined by flow cytometry (CytoFLEX; Beckman Coulter, Brea, CA, USA) and analyzed with FlowJo v7.6.1 software.

### Cell migration and invasion assays

Transwell assay was performed to evaluate cell migration and invasion abilities using Transwell chambers (CORNING Life Sciences, Corning, MA, USA). For the migration assay, 2 × 10^4^ transfected UM cells were suspended in a 200 μL serum-free medium in the upper chamber, while the lower chamber contained 800 μL medium with 10% fetal bovine serum. After 24 or 48 h (based on different cell lines), the upper chamber was washed, fixed, stained with 0.25% crystal violet, and counted under a light microscope. Cell numbers were counted in three random fields of view. For the invasion assay, the same steps were followed, with Matrigel (BD Biosciences, Franklin Lakes, NJ, USA) pre-coated onto the upper layer for 60 min at 25°C before the experiment.

For the wound healing assay, a 200-µL pipette tip was used to create a vertical scratch wound in the middle slide after cells were transfected and seeded at least 90% confluence into 6-well plates. Pictures of the wound were taken at the same position under a microscope at 0 and 24 h. Migration ability was analyzed by quantitatively evaluating the gap distance using ImageJ software.

### *Fluorescence* in situ *hybridization (FISH) and immunofluorescence*

RNA FISH was conducted to assess the subcellular localization of *circ_0053943* in MUM2B, C918, and OCM-1A cells using a FISH Kit (RiboBio) following the manufacturer’s instructions. *Circ_0053943*-specific Cy3-labeled probes were designed and synthesized by RiboBio. Briefly, cells were fixed with 4% paraformaldehyde, permeabilized with 0.5% Triton X-100 in phosphor-buffered saline (PBS), and blocked with a prehybridization buffer. Subsequently, the cells were incubated in a hybridization buffer containing a FISH probe overnight. After rinsing with sodium citrate buffer, cells were incubated with Hoechst 33342 (ThermoFisher Scientific, MA, USA) for nuclear staining. For dual RNA-FISH and immunofluorescence, cells were blocked with an immunostaining blocking solution (Beyotime) after incubation with the hybridization buffer containing the FISH probe. Subsequently, cells were incubated with primary antibodies overnight and labeled with fluorescence-conjugated secondary antibodies for 1 h in the dark. Hoechst 33342 (ThermoFisher Scientific) was added for nuclear visualization. Images were acquired using the Nikon A1Si Laser Scanning Confocal Microscope (Nikon Instruments, Tokyo, Japan).

### Western blotting

Tissue or cellular protein was lysed in RIPA lysis buffer (Beyotime) following the instructions, and the protein concentration was detected using the BCA kit (Vazyme, Nanjing, China). Western blotting experiments and signal quantification were performed according to the manufacturer’s protocols. Antibody information is provided in Suppl. Table 4.

### RNA pulldown assay

The biotin-labeled pulldown probe targeting *circ_0053943* and the negative control were designed and synthesized by RiboBio as detailed in Suppl. Table 5. The RNA-pulldown assay was conducted with the Pierce Magnetic RNA-Protein Pull-Down Kit according to the protocol (ThermoFisher Scientific). Lysates from UM cells were incubated with transcribed biotin-labeled *circ_0053943* and pulled down with streptavidin beads. Eluted proteins from the RNA pulldown assay were subjected to mass spectrometric analysis or western blot.

### Luciferase reporter assay

The luciferase reporter assay was employed to evaluate the direct binding specificity between *EGFR* 3′UTR and IGF2BP3. For the luciferase reporter assays, a wild (*EGFR*–3’UTR) or mutant (*EGFR*–3′UTR-Mut #1 or #2) fragment was inserted into a pmirGLO vector (Promega, Madison, WI, USA) containing the firefly luciferase gene (*hLuc+*) and renilla luciferase gene (*hRluc*). UM cells cultured in 24-well plates at 60%–80% confluency were transfected with the pmirGLO wildtype or mutant reporter vector along with *circ_0053943* plasmid or vectors using Lipofectamine 3000 (Invitrogen) according to the manufacturer’s protocol. The relative values of *hLuc+* and *hRluc* were detected by Centro LB960 XS3 (Berthold, Bad Wildbad, German), and each measurement was repeated three times.

### RNA sequencing

RNA-seq libraries were constructed in *circ_0053943*-silenced MUM2B cells. Initially, RNA integrity was assessed using the Agilent 2100 Bioanalyzer (Genesky, Shanghai, China) after quality inspection with starting RNA at 2 μg. Subsequently, Oligo-dT magnetic beads were employed for mRNA purification and fragmentation to 100–300 bp. Following this, first-and second-strand cDNA was synthesized, followed by end repair and adenylate 3′ ends. Finally, after ligating adapters, fragment size selection, and PCR amplification, a HiSeq system (Illumina, San Diego, CA, USA) in Pair End mode was used for high-throughput RNA sequencing. Differentially expressed genes (DEGs) between two groups were identified using the Deseq2 package in R v3.6.3. A cutoff criterion of the absolute value of fold change (|log2FC|) > 1 and *p*-value < 0.05 was selected for DEG identification.

### RNA immunoprecipitation (RIP)

A RIP Kit (Millipore, Burlington, MA, USA) was acquired for RIP assays. Initially, cells were lysed with RIP lysis buffer, along with protease and RNase inhibitors. Subsequently, magnetic beads were mixed with 10 μg anti-IGF2BP3, anti-Flag, or anti-IgG antibodies. Cell lysates were then mixed with magnetic beads and antibodies at 4°C overnight. Finally, the immunoprecipitated RNA was extracted for qRT-PCR after RNA purification.

### RNA stability assay

Relevant RNA level was measured by qRT-PCR after the extracted RNA of UM cells was incubated with or without RnaseR (3 U/μg) for 10 min at 37°C. For actinomycin D treatment, the total RNA of UM cells was harvested every 6 h after treatment (5 μg/mL). The variation trends of *circ_0053943* and *RasGRP3* were detected by qRT-PCR.

### Animal model

For the orthotopic and subcutaneous xenografted model, 1 × 10^6^ transfected cells were resuspended in PBS and subcutaneously injected into the armpits of BALB/c nude mice (four weeks old, male) obtained from the Animal Center of Nanjing Medical University (Nanjing, China) for the xenograft model. A total of 48 mice were randomly divided into eight research groups, each consisting of six mice. The tumor volume was calculated every week, and all mice were sacrificed after 4 weeks. Tumors were resected and fixed in formalin for subsequent immunohistochemistry detection.

For the tumor metastasis assay *in vivo*, 1 × 10^6^ relevant cells resuspended in 50 μL PBS were injected into the mice spleen via the left epigastric incision. A total of 40 mice were grouped into eight groups, with five mice per group. The livers of the mice were resected and fixed after 6 weeks. All animal experiments were performed according to the institutional guidelines of the Committee on the Ethics of Animal Experiments of Nanjing Medical University under the number IACUC-2108017.

### Statistical analysis

Every experiment was repeated at least three times. SPSS v29.0 software (Chicago, IL, USA) and GraphPad Prism software (La Jolla, CA, USA) were used for the statistical analyses. Before determining appropriate statistical tests or procedures, we conducted Shapiro-Wilk tests to detect the normality distribution of variables. Variation analysis of two groups was performed with Student’s *t*-test, while analysis of variance was used to analyze the difference between more than two groups.

## Results

### Verification of circ_0053943 in UM

**Figure 1:**
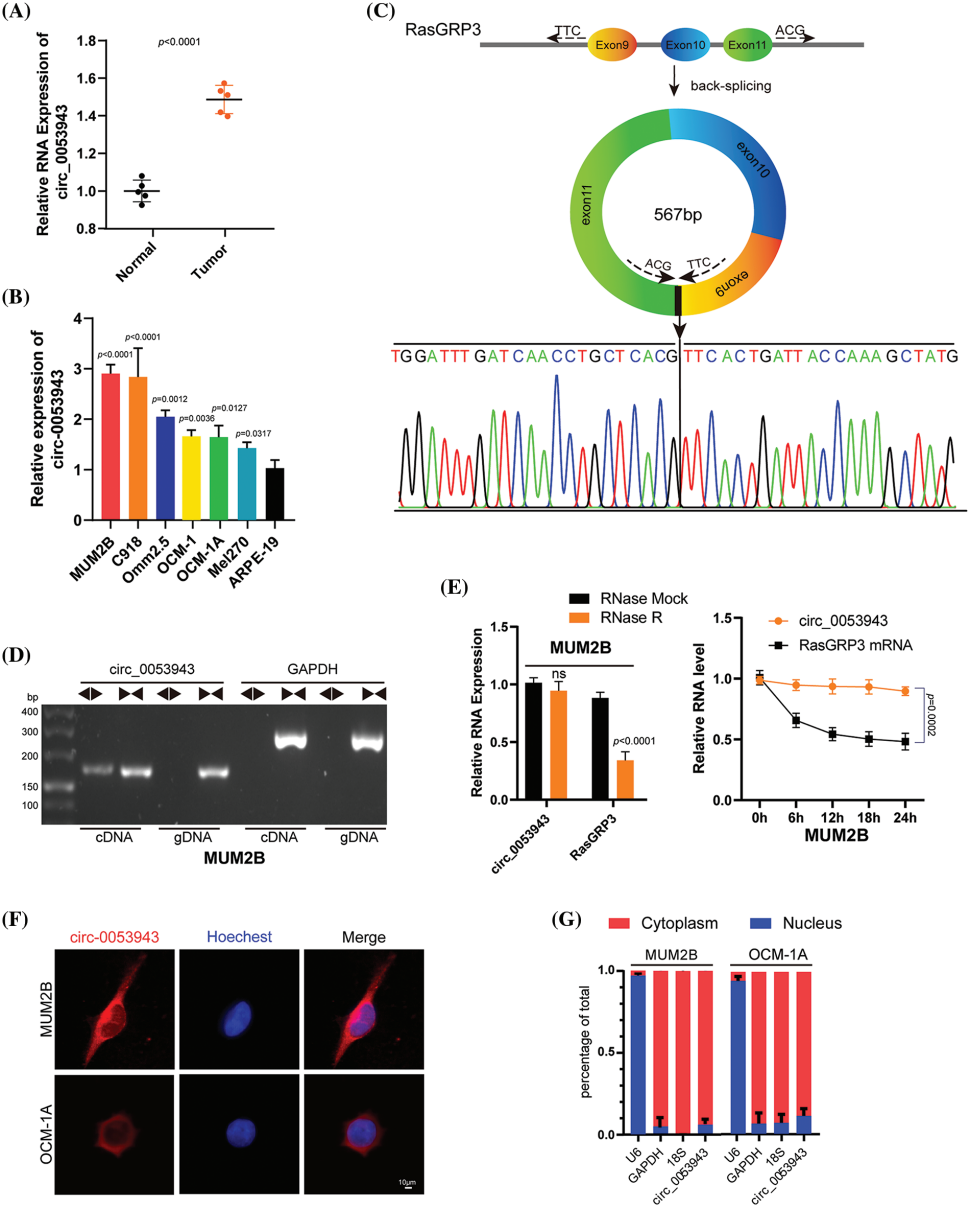
Characterization of *circ_0053943* in uveal melanoma (UM). (A) Expression of *circ_0053943* in 5 UM and 5 human normal melanocyte tissues in our cohort. (B) Expression of *circ_0053943* in human retinal pigment epithelial cell line (ARPE-19) and 6 UM cell lines. (C) The schematic illustration showed the back splicing of *circ_0053943*, and sanger sequence validated the splicing site. (D) PCR and agarose gel electrophoresis confirmed the circular formation of *circ_0053943*, using divergent and convergent primers in gDNA and cDNA of MUM2B cells. GAPDH was used as a negative control. (E) *Circ_0053943* and linear RasGRP3 expression levels were detected after RNase R in MUM2B. (F, G) RNA fluorescence *in situ* hybridization (FISH) (F) and subcellular fractionation assays. Scale bars, 50 μm. (G) Indicated that *circ_0053943* was predominately localized in the cytoplasm of UM cells. ^ns^*p* > 0.05.

The present study focused on upregulated circRNAs based on the published circRNA microarray data between UM and normal uveal tissues [12]. Then, quantitative reverse transcription-polymerase chain reaction (qRT-PCR) analysis was performed to detect these upregulated circRNAs in UM and normal tissues for further validation. *Circ_0053943* was identifeid as the most significantly differentially expressed circ RNAs upregulated in UM tissues (Fig. 1A), similar to the previously confirmed *circ_0119872* (Suppl. Fig. S1A) [12,13]. Subsequently, we assessed the level of *circ_0053943* in UM cell lines; as shown in Fig. 1B, its level in ARPE-19 cells was significantly lower than that in UM cell lines.

*Circ_0053943* originates from exons 9, 10, and 11 of *RasGRP3*, containing 567 nucleotides, according to the circBase annotation (Fig. 1C) [21]. To confirm its circular nature, convergent and divergent primers were designed to amplify linear and back-splicing products based on cDNA and genomic DNA (gDNA). Agarose gel electrophoresis demonstrated that convergent primers successfully amplified products of the expected size for *circ_0053943* and *GAPDH* from both cDNA and gDNA. However, divergent primers for *circ_0053943* only yielded a PCR product from cDNA, not gDNA (Fig. 1D and Suppl. Fig. S1B). Additionally, an RNase R treatment assay confirmed the higher resistance of *circ_0053943* to RNase R and actinomycin D treatment compared to linear *RasGRP3* mRNA (Fig. 1E and Suppl. Fig. S1C). RNA-FISH and RNA nucleus/cytoplasm separation revealed that *circ_0053943* was predominantly localized in the cytoplasm (Figs. 1F, 1G and Suppl. Figs. S1D–S1E). Three shRNAs targeting *circ_0053943* were transfected into MUM2B, Omm2.5, and C918 cells, while Mel270, OCM-1, and OCM-1A cells were developed with an overexpression lentivirus. Notably, the level of *RasGRP3* mRNA remained unchanged in cells with reduced or increased *circ_0053943* levels (Suppl. Figs. S1F, S1G).

### Circ_0053943 promotes the proliferation and metastasis of UM cells

**Figure 2:**
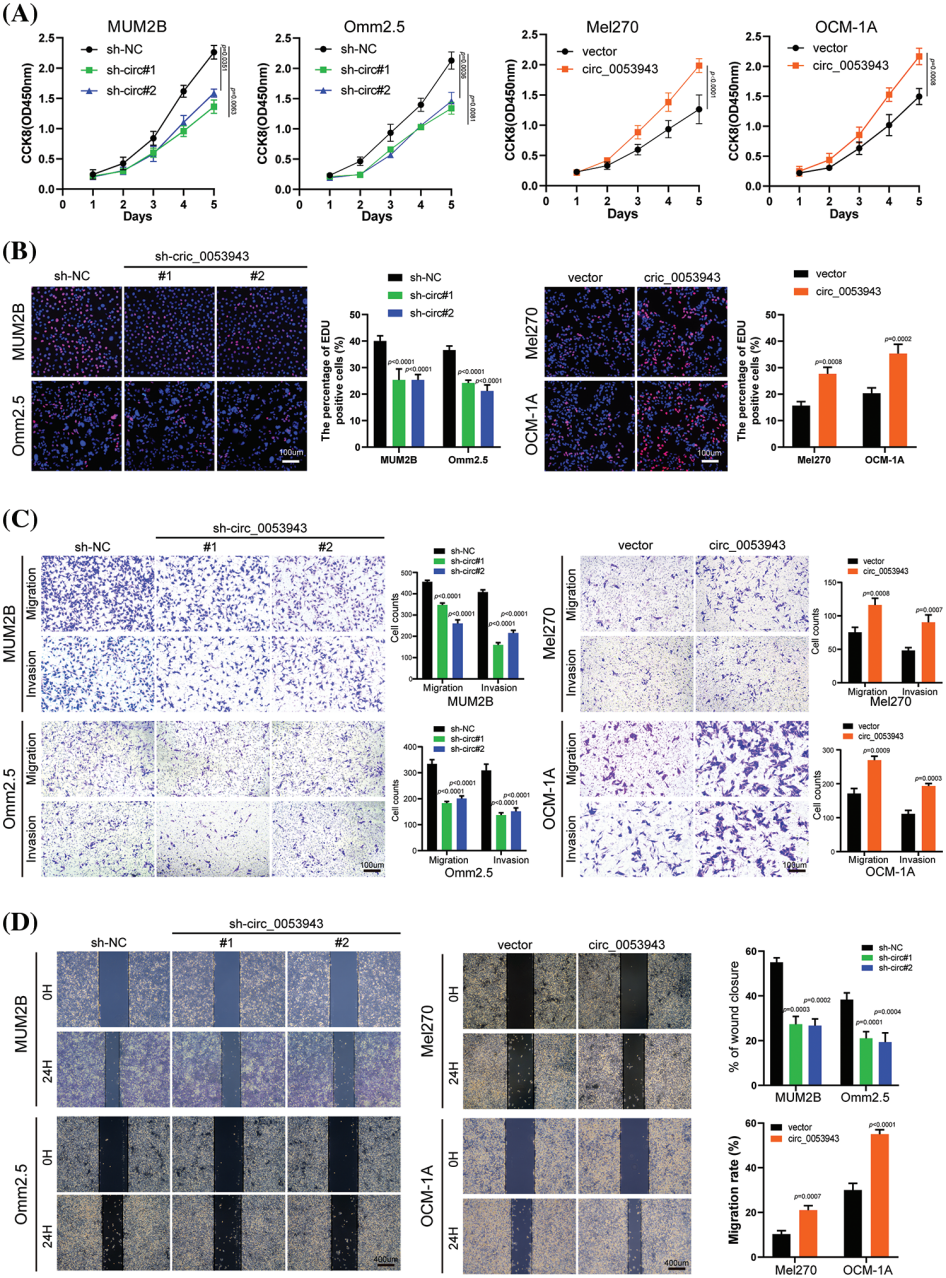
*Circ_0053943* promotes the proliferation, migration, and invasion of UM cells *in vitro*. (A) Cell Counting Kit-8 (CCK8) assays were applied to determine the growth curves of *circ_0053943* knockdown or overexpression cells. (B) 5-ethynyl-2-deoxyuridine (EDU) assays were performed to assess the cell proliferation ability. Scale bars, 100 μm. (C) Transwell migration and invasion assays were applied to evaluate the migration and invasion abilities of UM cells. Scale bars, 100 μm. (D) Cell migration ability was assessed by wound healing assay. Scale bars, 400 μm.

**Figure 3:**
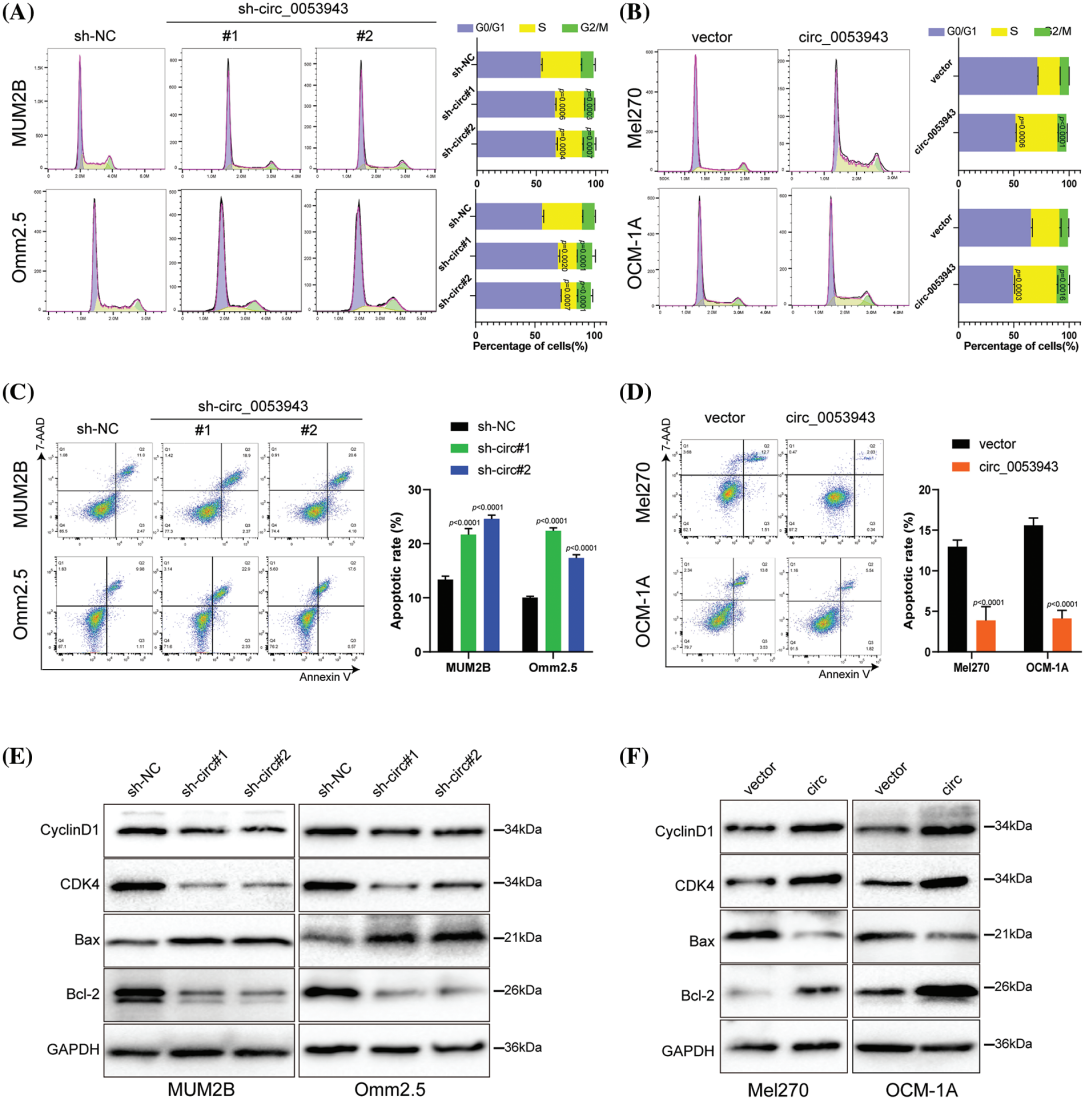
*Circ_0053943* regulates the cell cycle and apoptosis of UM cells. (A, B) Cell cycle distributions were detected by flow cytometry in *circ_0053943* knockdown or overexpression cells. (C, D) The apoptotic rates were performed and analyzed after cells were treated with 0.5 mM H_2_O_2_ for 4 h. All data are presented as the means ± SD of three independent experiments. (E, F) The expression of cell cycle and apoptosis makers (Cyclin D1, CDK4, Bcl-2, and Bax) were detected by western blot in relatively treated cells.

To elucidate the biological role of *circ_0053943* in UM, shRNA#1 and #2, along with a control shRNA, were chosen for cell phenotype assays due to their greater knockdown efficiency. Cell proliferation was assessed using the CCK8 and EDU staining assays. Downregulation of *circ_0053943* significantly suppressed UM cell proliferation, while upregulation of *circ_0053943* promoted cell proliferation (Figs. 2A, 2B and Suppl. Figs. S2A, S2B). Transwell assays and scratch wound healing assays revealed that *circ_0053943* knockdown significantly decreased tumor cell migration and invasion ability. In contrast, *circ_0053943* upregulation enhanced these abilities in UM cells (Figs. 2C, 2D and Suppl. Figs. S2C, S2D). Flow cytometric assays of cell cycle distribution indicated that downregulation of *circ_0053943* increased the percentage of cells in the G0/G1 phase and decreased the population in the S phase of the cell cycle (Fig. 3A). Conversely, upregulation of *circ_0053943* promoted the progression of G1-to-S phase transition remarkably in UM cells (Fig. 3B and Suppl. Fig. S2E). Apoptosis assay demonstrated that cells transfected with shRNAs exhibited higher apoptotic rates than the control, while transfection with the overexpressed lentivirus reduced apoptotic cells (Figs. 3C, 3D and Suppl. Fig. S2F). Western blot showed that reducing *circ_0053943* led to decreased protein levels of Cyclin D1, Cyclin-dependent kinase 4 (CDK4), and B-cell lymphoma 2 (Bcl-2) while increasing the level of Bcl2-associated X (Bax). These changes were consistent with the results obtained from flow cytometric assays. Conversely, the upregulation of *circ_0053943* had opposite effects (Figs. 3E, 3F and Suppl. Fig. S2G).

### Circ_0053943 and IGF2BP3 cooperate to play oncogenic roles

**Figure 4:**
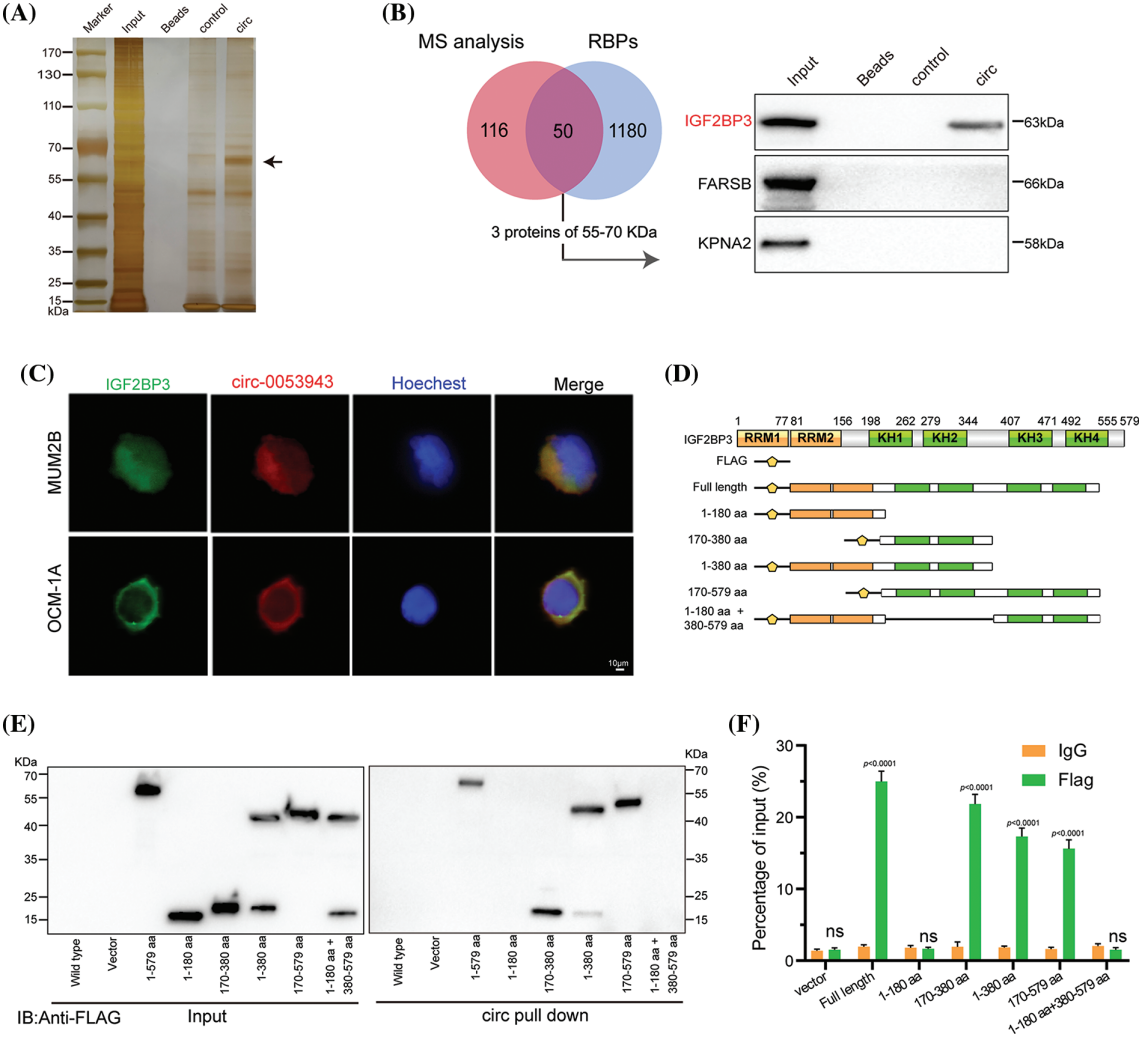
*Circ_0053943* binds to the KH1 and KH2 domains of IGF2BP3. (A, B) RNA pulldown assay followed by silver staining and western blot indicated that *circ_0053943* specifically interacted with IGF2BP3. (C) Dual RNA FISH and immunofluorescence assays showed that *circ_0053943* and IGF2BP3 colocalized in the cytoplasm of MUM2B and OCM-1A. Scale bars, 50 μm. (D) Functional domain and truncated mutation annotation of IGF2BP3. (E, F) RIP assay and RNA pulldown assay confirmed that *circ_0053943* characteristically interacted with the KH1 and KH2 domains of IGF2BP3. ^ns^*p* > 0.05.

The study explored the functional role of *circ_0053943* and its interaction with IGF2BP3 in UM progression. It was observed that *circ_0053943* may not act as a miRNA sponge in UM, as it was not significantly enriched by the Argonaute 2 (AGO2) antibody (Suppl. Fig. S3A**)**. To identify potential protein interactors of *circ_0053943*, an RNA pulldown assay coupled with mass spectrometry MS was conducted, revealing 167 differential proteins between the sense and antisense *circ_0053943* transcript pulldown groups in MUM2B cells (Suppl. Table 6). Among these proteins, 50 were identified as potential partners with RBP characteristics (Suppl. Table 7). Given the predominant cytoplasmic localization of *circ_0053943*, a biotin-labeled RNA pulldown assay using cytoplasmic protein extracts from MUM2B cells was performed. The sense-specific band at 55~70 kDa, obtained from sodium dodecyl sulfate-polyacrylamide gel electrophoresis, was excised for further analysis (Fig. 4A and Suppl. Fig. S3B). Among the proteins identified, only IGF2BP3 was detected in both the input group and *circ_0053943* pulldown products (Fig. 4B and Suppl. Fig. S3C). Furthermore, the study investigated the clinical relevance of IGF2BP3 levels in UM, revealing that patients with higher IGF2BP3 levels exhibited shorter overall survival and disease-free survival rates (Suppl. Fig. S3D). To confirm the interaction between *circ_0053943* and IGF2BP3, a RIP assay was performed using an anti-IGF2BP3 antibody. The results showed that the anti-IGF2BP3 antibody specifically enriched *circ_0053943* compared to the anti-IgG antibody (Suppl. Fig. S3E). Additionally, dual RNA-FISH and immunofluorescence assay demonstrated the co-localization of circ_0053943 and IGF2BP3 in MUM2B and OCM-1A cells, providing further evidence of their interaction (Fig. 4C).

Next, six truncated IGF2BP3 plasmids targeting its functional domains were designed. Protein domain mapping and RIP assay revealed that the KH1 and KH2 domains were essential for the interaction between IGF2BP3 and *circ_0053943* (Figs. 4D–4F). Moreover, modulation of *circ_0053943* level did not affect the mRNA and protein levels of IGF2BP3 (Suppl. Figs. S3F–S3G), and knockdown of *IGF2BP3* did not significantly alter the level of *circ_0053943* (Suppl. Figs. S3H–S3I). Importantly, IGF2BP3 depletion abolished the *circ_0053943*-induced cell proliferation, migration, and invasion. Conversely, upregulation of IGF2BP3 could not affect cell proliferation and metastatic ability when *circ_0053943* was knocked down (Suppl. Figs. S4A–S4F). These findings highlight the potential collaboration between *circ_0053943* and IGF2BP3 in driving UM oncogenesis.

### EGFR identified as a downstream target of circ_0053943 and IGF2BP3

**Figure 5:**
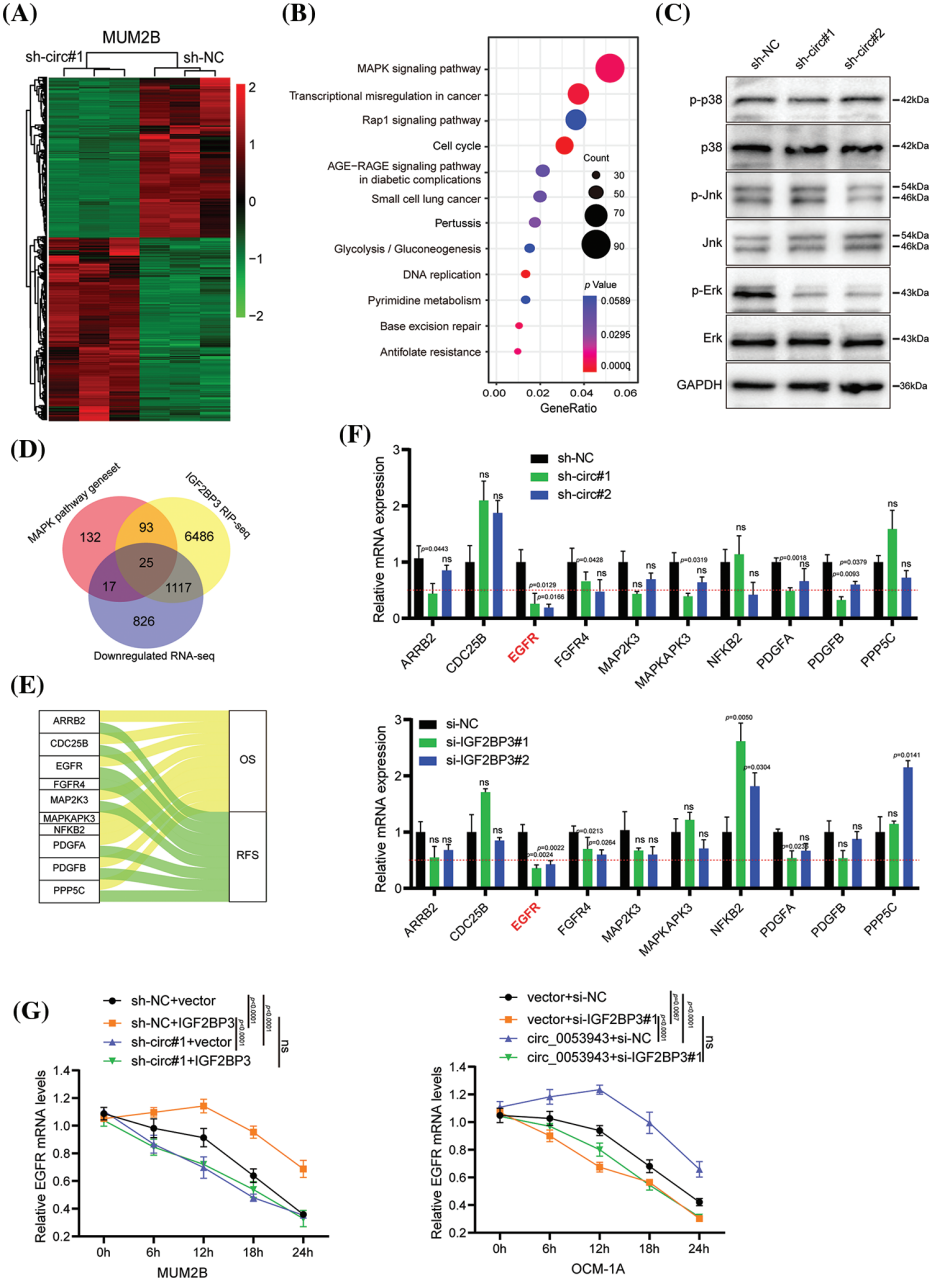
*EGFR* is downregulated by *circ_0053943* and correlated with UM progression. (A) Heatmap showed the RNA-seq result in *circ_0053943* knockdown and control MUM2B cells. (B) The top 12 enriched Kyoto Encyclopedia of Genes and Genomes (KEGG) terms for DEGs. (C) Western blot indicated total and phosphorylated proteins of MAPK signaling pathway. (D) Venn diagram showing the 25 overlapping genes by downregulated genes of silencing *circ_0053943* RNA-sequence data and MAPK pathway gene set together with IGF2BP3 binding genes of IGF2BP3 RIP-sequence data (GSE90642 and GSE90639). (E) Sankey diagram representing 10 overlapping genes associated with UM progression. (F) The transcript levels of these 10 genes in the *circ_0053943* knockdown (upper) and IGF2BP3 knockdown (down) MUM2B cells using qRT-PCR. (G) The half-life of *EGFR* mRNA after treatment with 5 μM actinomycin D for the indicated times in the silencing *circ_0053943* (left) and IGF2BP3 (right) cells with ectopically expressed IGF2BP3 and *circ_0053943*. All data are presented as the means ± SD of three independent experiments. ^ns^*p* > 0.05.

To elucidate the mechanism by which *circ_0053943* promotes UM progression through IGF2BP3, the study focused on the role of IGF2BP3 in stabilizing target mRNA transcripts, as previously reported in various cancers [22–24]. RNA-sequence analysis was performed to profile global gene expression changes upon *circ_0053943* knockdown in MUM2B cells. The analysis revealed a total of 4,293 dysregulated genes, including 2,308 upregulated and 1,985 downregulated genes (Fig. 5A). Pathway enrichment analysis based on the Kyoto Encyclopedia of Genes and Genomes (KEGG) database showed that the mitogen-activated protein kinase (MAPK) pathway was the most enriched (Fig. 5B). The key components of the MAPK pathway, including P38, extracellular signal-regulated kinase (ERK), c-Jun NH2-terminal kinase (JNK), and their phosphorylation forms, were evaluated using western blot. Interestingly, while the levels of P38, ERK1/2, and JNK did not show significant changes upon *circ_0053943* silencing, the phosphorylated forms of ERK significantly reduced (Fig. 5C).

A *circ_0053943* integrated analysis of the RNA-sequence data, two published IGF2BP3 RIP-sequence datasets [17], and critical components in the MAPK signaling pathway identified 25 potential target genes (Fig. 5D). Survival analysis of these genes by GEPIA 2 [24], revealed 10 genes that showed prognostic impacts in UM (Fig. 5E and Suppl. Figs. S5A–S5B). Validation in *circ_0053943* and IGF2BP3 knockdown cells showed that *ARRB2*, *EGFR*, *FGFR4*, *PDGFA*, and *PDGFB* exhibited decreased transcript levels upon *circ_0053943* depletion, with *EGFR* demonstrating a significant decrease of more than 50% in both *circ_0053943* and IGF2BP3 depletion cells (Fig. 5F).

EGFR was selected as the IGF2BP3-bound target altered by *circ_0053943* for further investigation. Western blot analysis confirmed that knockdown of either *circ_0053943* or *IGF2BP3* reduced the protein levels of EGFR (Suppl. Fig. S6A). Additionally, qRT-PCR and western blot demonstrated that knocking down *circ_0053943* abrogated the long-lasting effect of IGF2BP3 upregulation on the *EGFR* transcript. Conversely, reducing IGF2BP3 abolished the half-life, and the mRNA level increased by *circ_0053943* overexpression (Fig. 5G and Suppl. Figs. S6B, S6C). These findings suggested a functional interdependency between *circ_0053943* and IGF2BP3 in stabilizing the *EGFR* transcript, highlighting *EGFR* as a downstream target in the *circ_0053943*/IGF2BP3 axis contributing to UM progression.

### Circ_0053943 cooperates with IGF2BP3 to stabilize EGFR mRNA in an m6A-dependent manner

**Figure 6:**
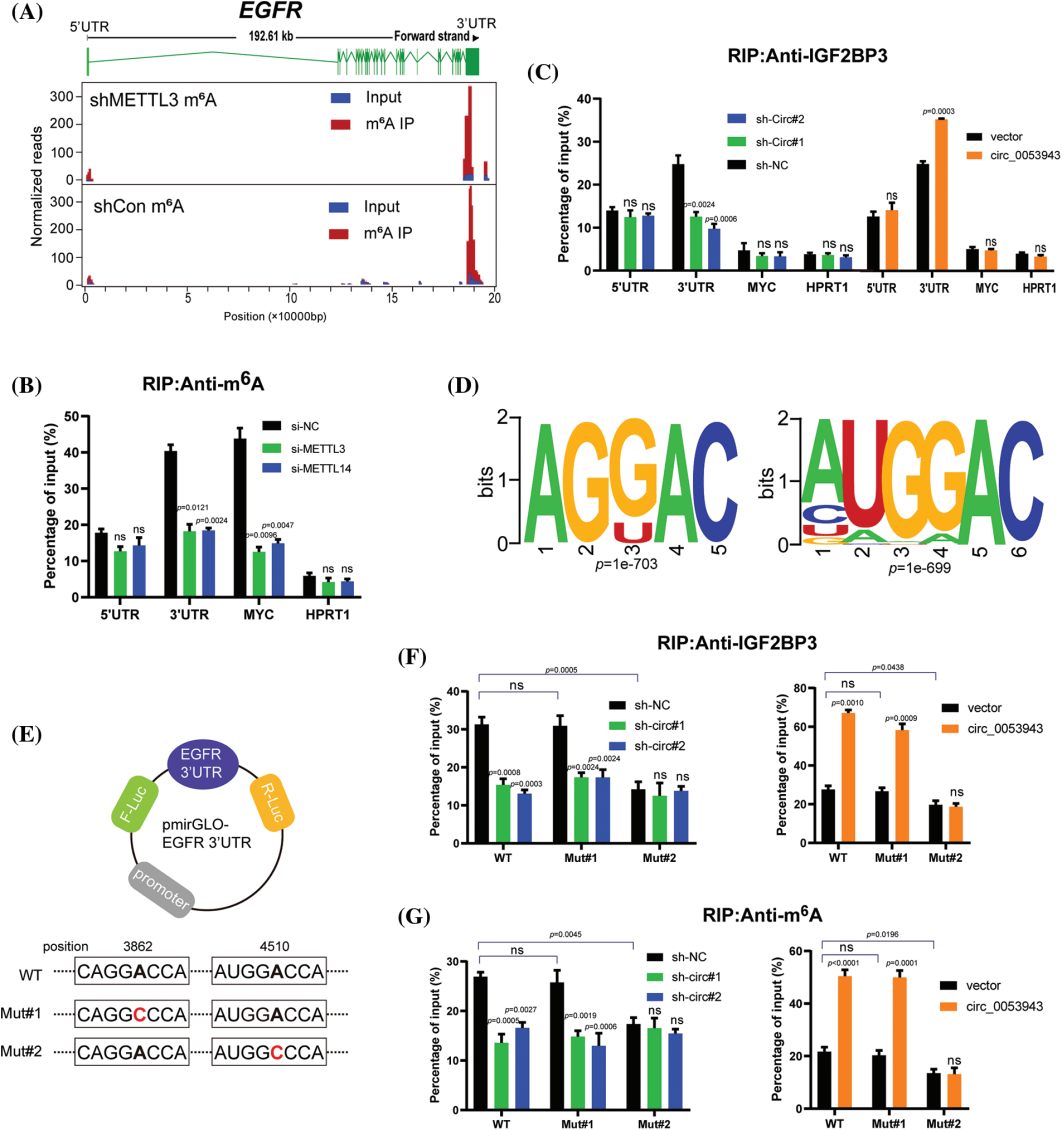
*Circ_0053943* cooperates with IGF2BP3 to regulate *EGFR* in an m^6^A-dependent manner. (A) Distribution of m^6^A peaks across the *EGFR* transcript based on m^6^A RIP-sequence data. (B) RIP qRT-PCR showing the enrichment of m^6^A modification in the *EGFR* 5′ UTR/3′ UTR regions in the METTL3 and METTL14 knockdown MUM2B cells. MYC CRD was a positive control. HPRT1 was a negative control. (C) RIP qRT-PCR detecting the enrichment of IGF2BP3 in the *EGFR* 3′UTR, 5′UTR, MYC CRD, and HPRT1 in *circ_0053943* knockdown and overexpressed cells. (D) Two top consensus sequences of IGF2BP3-binding sites and the m^6^A motif detected by SRAMPA and RMBase V2.0 motif analysis. (E) Schematic representation of wild-type (WT) and mutated (MUT) *EGFR* 3′UTR of the pmirGLO vector. (F, G) RIP qRT-PCR detection of the enrichment of IGF2BP3 and m^6^A in the *EGFR* 3′UTR WT and MUT luciferase reporters in the *circ_0053943* knockdown and overexpression cells. All data are presented as the means ± SD of three independent experiments. ^ns^*p* > 0.05.

To investigate whether *circ_0053943* and IGF2BP3 regulate the stability of *EGFR* mRNA in an m^6^A-dependent manner, the study identified potential m^6^A-modified regions of *EGFR* using m^6^A RIP-sequence data. The canonical “GGAC” m^6^A motif was found in these m^6^A peaks, confirming that *EGFR* mRNA is indeed modified by m^6^A methylation (Fig. 6A).

M6A-specific RIP was then performed to determine the m^6^A methylation level of *EGFR* in METTL3 and METTL14 knockdown cells (Suppl. Fig. S7A). A reduction in the coding region instability determinant (CRD) of MYC (positive control), but not in HPRT1 (negative control), confirmed that *EGFR* was modified by m^6^A (Fig. 6B). Since IGF2BP3 preferentially binds to the “GGAC” m^6^A core motif of its targets [17], the study examined whether *circ_0053943* affects the binding of IGF2BP3 to the m^6^A-modified *EGFR*. IGF2BP3 RIP qRT-PCR assays showed that knockdown of *circ_0053943* significantly reduced the binding of IGF2BP3 to the m^6^A site in the 3′UTR region of *EGFR*, while overexpression of *circ_0053943* facilitated this binding. This effect was not observed in the *EGFR* 5′UTR or MYC CRD (Fig. 6C). Moreover, an RNA pulldown assay with *in vitro*-transcribed *circ_0053943* confirmed that the binding ability of *circ_0053943* to the 3′UTR of *EGFR* was largely abolished following *IGF2BP3* knockdown and increased after *IGF2BP3* overexpression (Suppl. Fig. S7B). These findings confirm that *circ_0053943* may bind to the 3′UTR of *EGFR* in an IGF2BP3-dependent manner.

In the *EGFR* 3′UTR region, two “GGAC” m^6^A motifs were predicted by m^6^A modification site predictors SRAMPA and RMBase v2.0 (Fig. 6D and Suppl. Table 8). Luciferase reporters containing the wild-type *EGFR* 3′UTR (WT-3′UTR), mutant #1, and #2 3′UTR (Mut#1-and Mut#2-3′UTR; changing GGAC to GGCC) were constructed to elucidate the potential roles of *circ_0053943* in m^6^A modification of *EGFR* (Fig. 6E). Both IGF2BP3 and m^6^A RIP qRT-PCR assays showed higher enrichment with the WT and Mut#1-3′UTR reporters compared to Mut#2-3′UTR. Overexpression of *circ_0053943* or IGF2BP3 remarkably increased the binding of IGF2BP3 and m^6^A in the WT and Mut#1-3′UTR but not in the Mut#2-3′UTR report. Conversely, knocking down *circ_0053943* and IGF2BP3 independently reduced this binding except in the Mut#2-3′UTR report (Figs. 6F, 6G). Furthermore, luciferase reporter assays demonstrated that the relative luciferase activities were decreased by knockdown of *circ_0053943* or IGF2BP3 in the WT and Mut#1-3′UTR but not in Mut#2-3′UTR reporter. Similarly, the relative luciferase activity of MUT#2-3′UTR reporter was also not altered by upregulated *circ_0053943* or IGF2BP3 (Figs. 6H, 6I). Thus, methylation of the m^6^A-modified site c.4510A in *EGFR* 3′UTR contributed to *EGFR* expression.

### Circ_0053943 promotes UM proliferation, metastasis, and angiogenesis through the upregulation of EGFR in vitro

**Figure 7:**
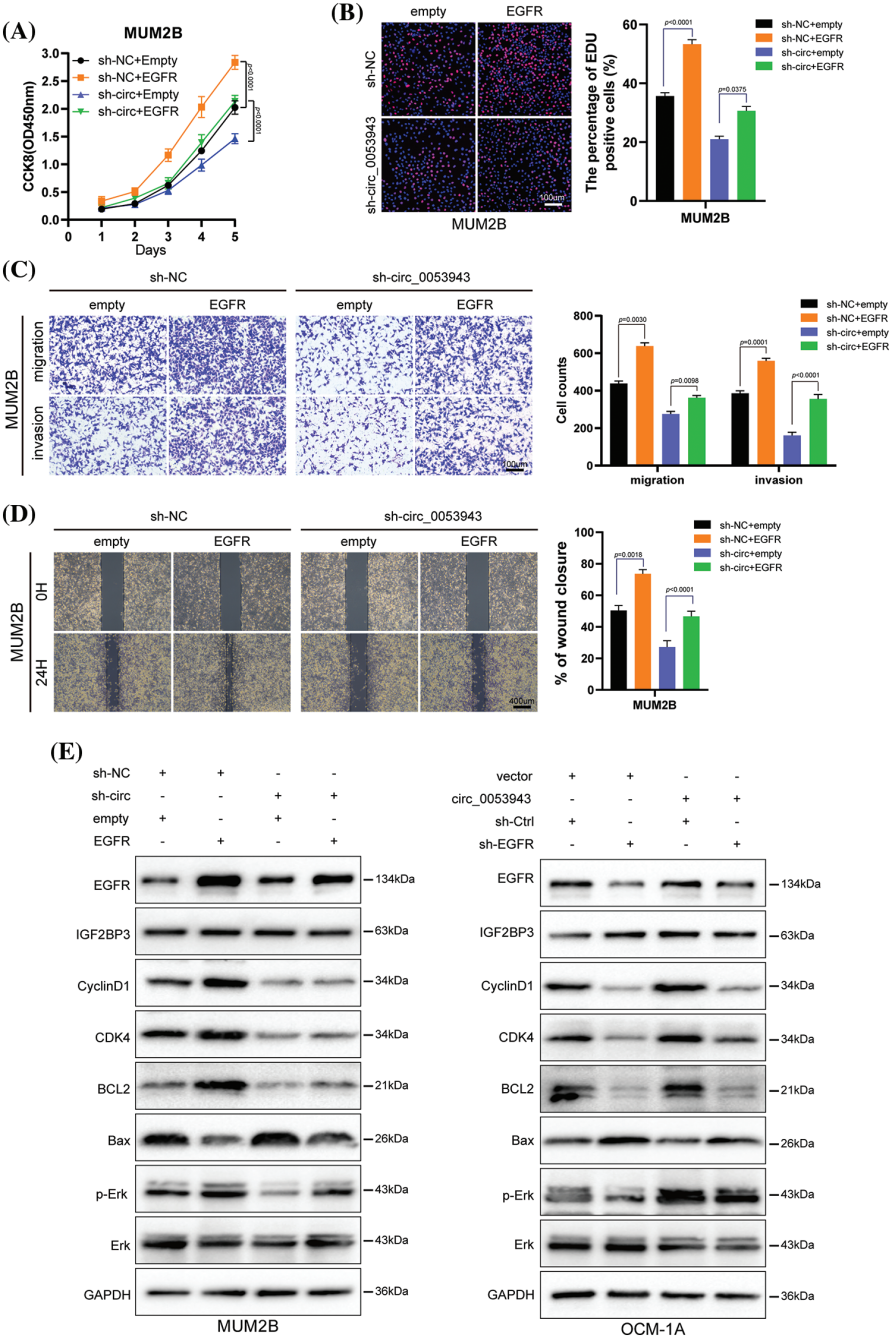
*Circ_0053943* regulates UM proliferation, metastasis, cell cycle, and apoptosis via upregulating *EGFR* expression *in vitro*. (A, B) CCK8 and EdU were used to detect UM cell proliferation ability in *circ_0053943* knockdown cells with ectopically expressed *EGFR*. Scale bars, 100 μm. (C, D) Transwell and wound healing assays detected UM cell metastasis capacity in *circ_0053943* knockdown cells with ectopically expressed *EGFR*. Scale bars, 100 μm (C), 400 μm (D). (E) The expression of cell cycle and apoptosis makers (Cyclin D1, CDK4, Bcl-2, and Bax) along with *EGFR*, IGF2BP3, Erk, and p-Erk were detected by western blot in relatively treated cells. All data are presented as the means ± SD of three independent experiments.

*EGFR*-specific shRNA (sh-*EGFR*) and the negative control (sh-Ctrl), as well as an *EGFR* overexpression plasmid, were transfected into UM cells (Suppl. Fig. S8A). Cell proliferation assays revealed that *EGFR* silencing significantly inhibited the proliferation ability of *circ_0053943* upregulated cells. Conversely, *EGFR* overexpression promoted UM cell proliferation in *circ_0053943* knockdown cells (Figs. 7A, 7B, and Suppl. Figs. S8B, S8C). Transwell assays and wound healing assays indicated that metastatic abilities of *circ_0053943* knockdown cells were significantly increased in sh-*EGFR* cells compared to sh-Ctrl cells (Figs. 7C, 7D and Suppl. Figs. S8D, S8E).

Considering the role of *EGFR* in tumor angiogenesis, the potential implication of angiogenesis was analyzed using the HUVEC tube formation assay. Conditioned medium (CM) from sh-*circ_0053943* tumor cells dramatically reduced the formation of capillary-like structures by HUVECs. Conversely, *EGFR* overexpression reversed the decreased capillary-like structure formation caused by *circ_0053943* depletion. Meanwhile, CM from *circ_0053943* UM cells showed promoting effects on capillary-like structure formation, which was inhibited by silencing *EGFR* (Suppl. Figs. S8F, S8G).

Western blotting results showed that the knockdown of *circ_0053943* impaired the G1 to S transition by downregulating cycle-related proteins (Cyclin D1, CDK4), and the overexpression of *EGFR* could rescue it. *Circ_0053943* upregulation promoted the G1 to S transition, while silencing *EGFR* impaired this transition. Alterations in apoptosis-related proteins (Bcl-2, Bax) demonstrated that the anti-apoptotic effect of *circ_0053943* was weakened upon *EGFR* knockdown (Figs. 7E, 7F).

### Circ_0053943 promotes UM proliferation, metastasis and angiogenesis through upregulating EGFR in vivo

To investigate the effects of *circ_0053943* on UM proliferation and metastasis *in vivo*, subcutaneous xenograft nude mice models were established. MUM2B and OCM-1A cells transfected with sh-*circ_0053943*/sh-*EGFR*/overexpression plasmids, along with the respective control group and co-transfection group, were separately injected into nude mice.

**Figure 8:**
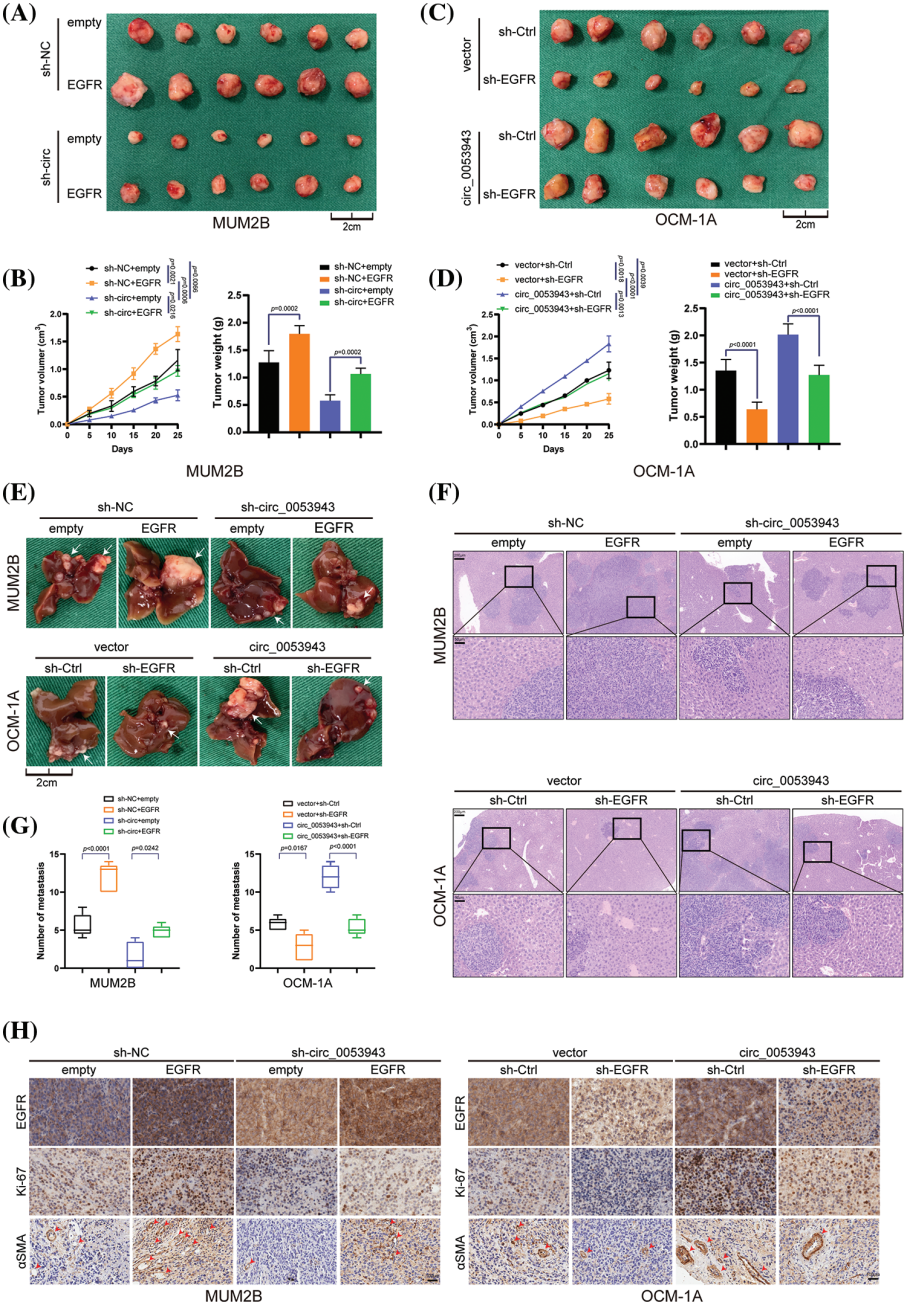
*Circ_0053943* promotes UM proliferation and metastasis via upregulating *EGFR* expression *in vivo*. (A, B) Representative photographs of subcutaneous xenograft tumors were obtained from the different groups of nude mice transfected MUM2B cells with knockdown *circ_0053943* and ectopically expressed *EGFR*. Tumors were observed by tumor size and average weight. (C, D) Representative photographs of subcutaneous xenograft tumors were obtained from the different groups of nude mice transfected OCM-1A cells with knockdown *EGFR* and ectopically expressed *circ_0053943*. Tumors were observed by tumor size and average weight. (E–G) Representative photographs (E) and HE staining (F) of liver metastases were obtained from nude mice transfected with relatively treated cells, and the number of metastases (G) was measured. Scale bars, 200 μm (up), 50 μm (down). (H) Protein levels of *EGFR*, Ki-67 and α-SMA in the tumor samples were determined by IHC. All data are presented as the means ± SD of three independent experiments. Scale bars, 100 μm.

Results showed that tumor growth was repressed by *circ_0053943* knockdown, with smaller tumor volume and weight compared to the control groups. Overexpression of *EGFR* reversed the growth inhibition caused by *circ_0053943* depletion (Figs. 8A, 8B). In *circ_0053943* upregulated cells, the opposite effects were observed due to the repression by *EGFR* depletion (Figs. 8C, 8D).

Anatomical results confirmed that metastatic nodules were reduced in the *circ_0053943* knockdown group compared with the control group. Conversely, metastatic lesions at the liver surface were more abundant in the *EGFR* overexpression group. Further analysis showed that the reduction of metastatic nodules caused by *circ_0053943* depletions was increased by *EGFR* overexpression. In contrast, the opposite effect was observed in nude mice injected with *circ_0053943*/sh-*EGFR* cells (Figs. 8E–8G).

Immunohistochemistry results demonstrated that levels of EGFR, Ki-67 (indicating tumor proliferation), and α-SMA (indicating angiogenesis) were decreased in the *circ_0053943* depletion group and increased in the overexpression group (Fig. 8H).

Collectively, these results confirmed that *circ_0053943* upregulation enhanced the proliferation, metastasis, and angiogenesis capacity of UM by upregulating *EGFR in vivo*.

## Discussion

The present study has demonstrated the crucial oncogenic role of *circ_0053943*, a highly expressed circRNA in UM cells. Our phenotype experiment reveals that *circ_0053943* promotes proliferation and metastasis in UM, including proliferation, migration, invasion, and angiogenesis of UM cells *in vitro* and tumorigenesis and hepatic metastasis *in vivo*. Notably, our study unveils a novel regulatory mechanism of *circ_0053943* in promoting UM progression through its interaction with a protein. Specifically, increased *circ_0053943* cooperates with IGF2BP3, forming a *circ_0053943*/IGF2BP3/*EGFR* mRNA-protein ternary complex. Consequently, it enhances the stability of *EGFR* mRNA, leading to the upregulation of *EGFR* and activation of MAPK/ERK signaling pathways in UM.

While cutaneous melanoma and UM share some similarities, it is essential to note that UM exhibits minimal overlapping genetic signatures with cutaneous melanoma [7,25,26]. In UM, genetic aberrancies are believed to drive tumor carcinogenesis through the abnormal activation of the Gα11/Q pathway [27,28]. Specifically, genes including *GNAQ*, *GNA11*, *PLCB4*, and *CYSTLR2*, and three secondary driver genes (*BAP1*, *SF3B1*, *EIF1AX*) have been reported to be frequently mutated in a substantial fraction of UM tissues [27,29–31]. However, somatic mutations above were observed only in limited UM cases, and *GNAQ* and *GNA11* are relatively weak oncoproteins without co-mutated secondary driver genes [32]. Previous studies have emphasized the importance of aberrant ncRNA expressions in oncogenesis, providing a unique opportunity to optimize treatment paradigms and establish new therapeutic options for UM [33–36]. Therefore, further studies on the pathological functions of ncRNAs are required to obtain a complete picture of the undeveloped regulatory mechanisms under these somatic mutations.

CircRNAs represent a novel class of endogenous ncRNAs characterized by a covalently closed loop structure. Accumulating evidence suggests that dysregulated circRNA is associated with clinical and pathological features in various tumors, confirming its potential biological significance [37,38]. To date, only two studies have investigated the dysregulation of circRNAs in UM. Yang et al. identified an abnormal expression profile of circRNAs in UM tissues but did not explore the underlying mechanisms [12]. Building on these identified circRNAs, Liu et al. demonstrated that upregulated cytoplasmic *circ_0119872* could promote UM tumorigenesis by acting as a miRNA sponge, modulating the activity of miRNAs on target genes [14]. Interestingly, *circ_0053943*, investigated in this study, was also predominantly located in the cytoplasm of UM cells, suggesting a potential link to post-transcriptional modifications. Moreover, it was unexpectedly found to be unable to bind with AGO2, ruling out the possibility of functioning as a miRNA sponge. To date, other mechanisms of circRNAs in tumor progression, particularly in UM, have not been thoroughly explored. This study identified that *circ_0053943* could interact with the KH1 and KH2 domains of IGF2BP3, enhancing its biological function.

Insight into the functions of IGF2BPs (IGF2BP1/2/3) as a newly discovered family of the m^6^A “reader,” regulating mRNA stability and translation, has grown [39,14]. Despite several findings demonstrating the critical role of m^6^A modification in UM tumorigenesis, little is known about the effects of IGF2BPs in this disease [40,41]. Wan et al. reported widespread upregulation of IGF2BP3 in various ocular cancers, including UM [42]. Survival analysis using The Cancer Genome Atlas further indicates that IGF2BP3 can independently predict prognosis for patients with UM. Our findings suggest a latent mechanism by which IGF2BP3 facilitates tumor development, and *circ_0053943* enhances this function in UM. Mechanistically, under the control of *circ_0053943*, IGF2BP3 directly binds to the m^6^A site, forming a *circ_0053943*/IGF2BP3/*EGFR* mRNA-protein ternary complex that enhances mRNA stability or translation of *EGFR* in an m^6^A-dependent manner. However, the molecular mechanism underlying IGF2BP3 upregulation in UM remains enigmatic.

*EGFR*, a 170-kDa transmembrane tyrosine kinase receptor, is detected in various malignant cells and has been reported to play a vital role in tumor development and progression [43]. Additionally, *EGFR* has been reported to regulate various downstream signaling pathways, including phosphatidylinositol-3-kinases/protein kinase B (PI3K/Akt) and MAPK/ERK signaling pathways [44–47]. Accumulating evidence shows that *EGFR* upregulation is significantly associated with tumor development and indicates poor prognosis in UM [48,49]. However, most studies have primarily focused on verifying how the upregulation of *EGFR* affects cancer cell proliferation and migration. This, however, is only part of the story, as the cause of *EGFR* upregulation in UM has not been thoroughly investigated. Therefore, our study demonstrated that upregulated *circ_0053943* could enhance *EGFR* expression through the *circ_0053943*/IGF2BP3/*EGFR* complex, ultimately activating the MAPK/ERK pathway. These findings were consistent with a previous study [50] and offered insights into possible upstream regulators.

Our study provides evidence that *circ_0053943* and IGF2BP3 cooperatively regulate the m^6^A modification of *EGFR* in UM, shedding new light on post-transcriptional *EGFR* expression and emphasizing the essential role of circRNAs in RNA metabolism. However, the mechanisms that upregulate *circ_0053943* and mediate its export *circ_0053943* from the nucleus to the cytoplasm require further investigation. Additionally, we did not determine how *circ_0053943* mediated the interaction between IGF2BP3 and *EGFR* by specifically binding to the KH1 and KH2 domains of IGF2BP3. RNA-seq and subsequent qRT-PCR verification demonstrated that the function of *circ_0053943* on IGF2BP3-modified target mRNA is selective. The specificity of ncRNA function remains elusive, but it aligns with a recent study based on clear cell renal cell carcinoma [51]. Furthermore, it is important to note that the published IGF2BP3 RIP-sequence data mentioned in this study are not from UM cell lines [17]. Therefore, the identification of the IGF2BP3 binding molecular spectrum based on UM cells needs further exploration. Another strength of our study is that we substantiated our results using an *in vivo* metastasis animal model. While the animal model may not fully replicate the complexity of UM in humans, our findings from this model partly support the clinical data and *in vitro* function of *circ_0053943* in promoting UM development. To date, the use of MAPK/ERK kinase (MEK) inhibitors in the management of metastatic uveal melanoma remains controversial, considering inevitable side effects [51,52].

## Conclusions

In summary, we identified a novel circRNA, *hsa*_*circ_0053943*, overexpressed in UM and associated with a poor prognosis. Upregulating *circ_0053943* exerted effect in stabilizing *EGFR* mRNA by forming a *circ_0053943*/IGF2BP3/*EGFR* RNA-protein ternary complex, which finally promotes the proliferation and aggressiveness of UM cells. Overall, our finding may provide a new target, *circ_0053943*, for the diagnosis and treatment of UM.

## Supplementary Materials

FIGURE S1 The screening of circRNAs and validation of circ_0053943 knockdown and overexpression. (A) The expression of has_circ_0119872 in 5 ocular melanoma tissues and 5 human normal melanocyte tissues in our cohort. (B) PCR and agarose gel electrophoresis confirmed the circular formation of circ_0053943, using divergent and convergent primers in gDNA and cDNA of C918 cells. GAPDH was used as a negative control. (C) Circ_0053943 and linear RasGRP3 expression levels were detected after actinomycin D treatment in C918. (D, E) RNA fluorescence in situ hybridization (FISH) (D) and Subcellular fractionation assays (E) indicated that circ_0053943 was predominately localized in the cytoplasm of UM cells. Scale bars, 50 μm (F) The efficiency of circ_0054943 knockdown in MUM2B, C918 and Omm2.5. The expression of RasGRP3 mRNA remained unchangeable. (G) The efficiency of circ_0053943 overexpression in OCM-1, OCM-1A and Mel270. The expression of RasGRP3 mRNA level was detected at the same time. All data are presented as the means ± SD of three independent experiments. ^ns^*p* >0.05

FIGURE S2 Circ_0053943 regulates UM cell proliferation, migration, invasion, cell cycle and apoptosis in vitro. (A) CCK8 assays were applied to determine the growth curves of circ_0053943 knockdown or overexpression cells. (B) EdU assays were performed to assess the cell proliferation ability. (C) Transwell migration and invasion assays were applied to evaluate the migration and invasion abilities of UM cells. Scale bars, 100 μm (D) Cell migration ability was assessed by wound healing assay. Scale bars, 400 μm (E) Cell cycle distributions were detected by flow cytometry in circ_0053943 knockdown or overexpression cells. (F) The apoptotic rates were performed and analyzed after cells were treated with 0.5 mM H2O2 for 4 h. All data are presented as the means ± SD of three independent experiments. (G) The expression of cell cycle and apoptosis makers (Cyclin D1, CDK4, Bcl-2, and Bax) were detected by western blot in relatively treated cells. All data are presented as the means ± SD of three independent experiments.

FIGURE S3 Circ_0053943 could specifically interact with IGF2BP3. (A) Analysis for circ_0053943 and ciRS-7 enrichment, relative to IgG. RIP assays were performed using FLAG antibody in MUM2B cells transfected with FLAG-AGO2 plasmid. (B, C) RNA pulldown assay followed by silver staining (B) and MS (C) indicated that circ_0053943 specifically interacted with IGF2BP3. (D) Kaplan-Meier plots indicated the OS and DFS of IGF2BP3 in UM patients from TCGA. (E) RNA pulldown assay followed by RIP assay indicated that circ_0053943 specifically interacted with IGF2BP3. (F, G) The transcript (F) and protein (G) level of IGF2BP3 in circ_0053943 knockdown and overexpression cells. (H) The knockdown and overexpression efficiency of IGF2BP3. (I) The circ_0053943 expression level in IGF2BP3 silencing and upregulated cells. All data are presented as the means ± SD of three independent experiments. ^ns^*p* >0.05.

FIGURE S4 Circ_0053943 and IGF2BP3 cooperative to play oncogenic roles. (A-F) EdU (A, B), Transwell (C, D), and wound healing assays (E, F) were used to detect UM cell proliferation ability in circ_0053943 knockdown cells with ectopically expressed IGF2BP3 and knockdown cells with ectopically expressed circ_0053943. Scale bars, 100 μm (A-D), 400 μm (E, F). All data are presented as the means ± SD of three independent experiments. ^ns^*p* >0.05.

FIGURE S5 The screening of genes connected with UM progression. (A, B) The survival map (A) for 25 overlapping genes in UM was analyzed by the GEPIA website tool (Mantel-Cox test) based on the TCGA database. *p*< 0.05 was framed and further showed in Kaplan-Meier plots (B).

FIGURE S6 Circ_0053943 and IGF2BP3 cooperative to regulate EGFR. (A) The protein levels of EGFR in circ_0053943 and IGF2BP3 knockdown cells. (B) The mRNA levels of EGFR in circ_0053943 knockdown cells with ectopically expressed IGF2BP3 (left), and IGF2BP3 knockdown cells with ectopically expressed circ_0053943 cells (right). (C) The protein levels of EGFR in circ_0053943 knockdown cells with ectopically expressed IGF2BP3 (left), and IGF2BP3 knockdown cells with ectopically expressed circ_0053943 cells (right). All data are presented as the means ± SD of three independent experiments. ^ns^*p* >0.05.

FIGURE S7 Circ_0053943 cooperates with IGF2BP3 to regulate EGFR in an m6A-dependent manner. (A) The knockdown efficiency of METTL3 and METTL14. (B) RIP qRT-PCR detecting the enrichment of biotin-labeled circ_0053943 in the EGFR 3'UTR, 5’UTR, MYC CRD, and HPRT1 in IGF2BP3 knockdown and overexpressed cells. (C, D) Relative luciferase activity and luciferase mRNA expression of the luciferase reporter gene with EGFR 3'UTR WT and MUT reporters in control and circ_0053943 knockdown or overexpression MUM2B cells. All data are presented as the means ± SD of three independent experiments. ^ns^*p* >0.05.

FIGURE S8 Circ_0053943 regulates UM proliferation, metastasis, and angiogenesis via upregulating EGFR expression in vitro. (A) The knockdown and overexpression efficiency of EGFR. (B, C). CCK8 (B) and EdU (C) were used to detect UM cell proliferation ability in circ_0053943 overexpression cells with silencing EGFR. Scale bars, 100 μm. Transwell (D) and wound healing assays (E) detected UM cell metastasis capacity in circ_0053943 overexpression cells with silencing EGFR. Scale bars, 100 μm (D), 400 μm (E). (F, G) Tube formation assay of HUVECs treated with conditional medium (CM) derived relatively transfected MUM2B (F) and OCM-1A (G). Scale bars, 400 μm. All data are presented as the means ± SD of three independent experiments.

## Data Availability

All data supporting the conclusions of this study have been included in this article. Please contact the authors for requests for raw data.

## Supplementary Materials

The supplementary material is available online at https://doi.org/10.32604/or.2023.045972.
