# Supplementary figures and images for: Circ_0053943 complexed with IGF2BP3 drives uveal melanoma progression via regulating N6-methyladenosine modification of Epidermal growth factor receptor
Source: Oncol Res. 2024 Apr 23;32(5):983–98. doi: 10.32604/or.2024.045972 (PMC11055987; doi:10.32604/or.2024.045972)

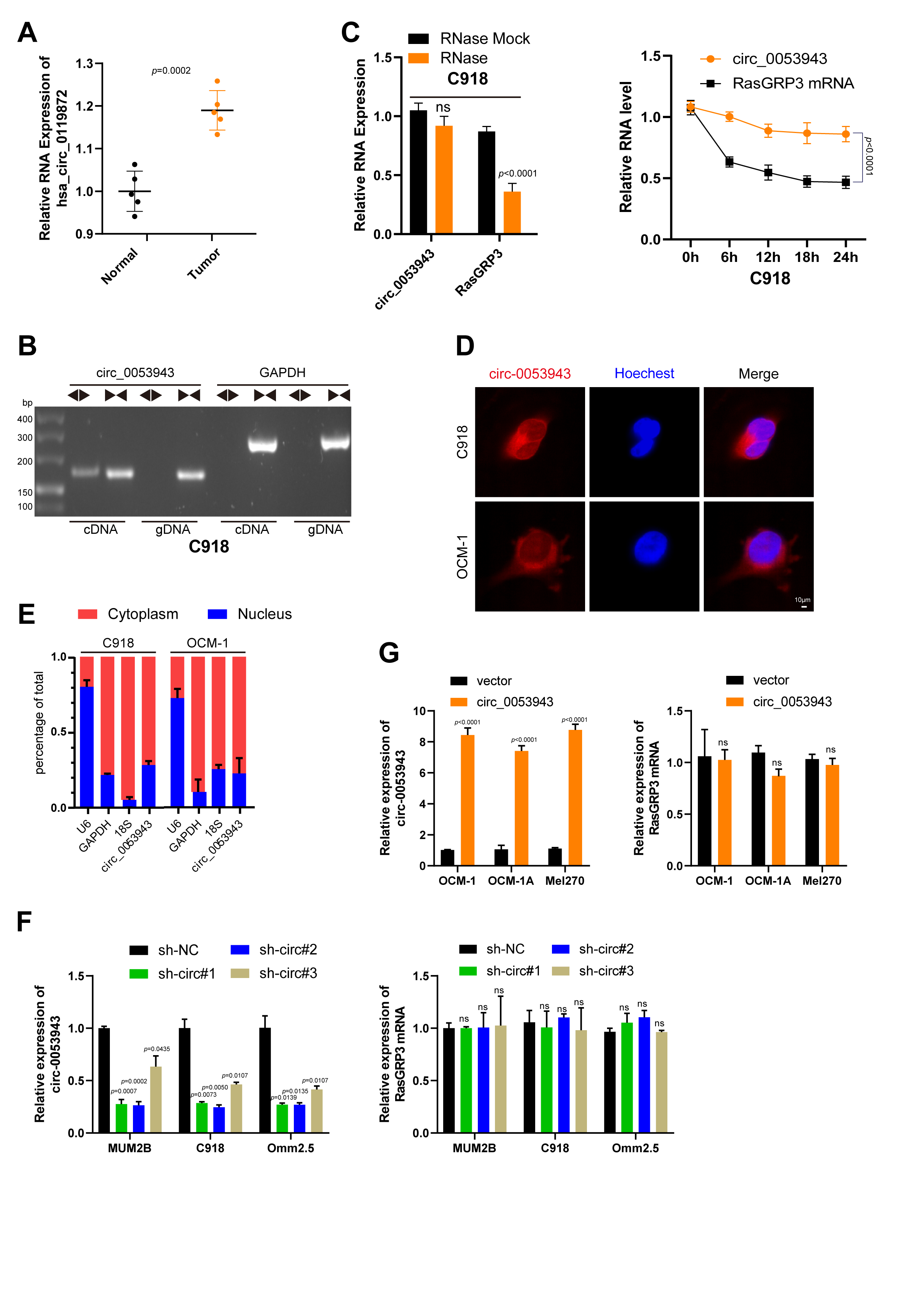

Supplement: FIGURE S1 [file OncolRes-32-45972-s001.tif]

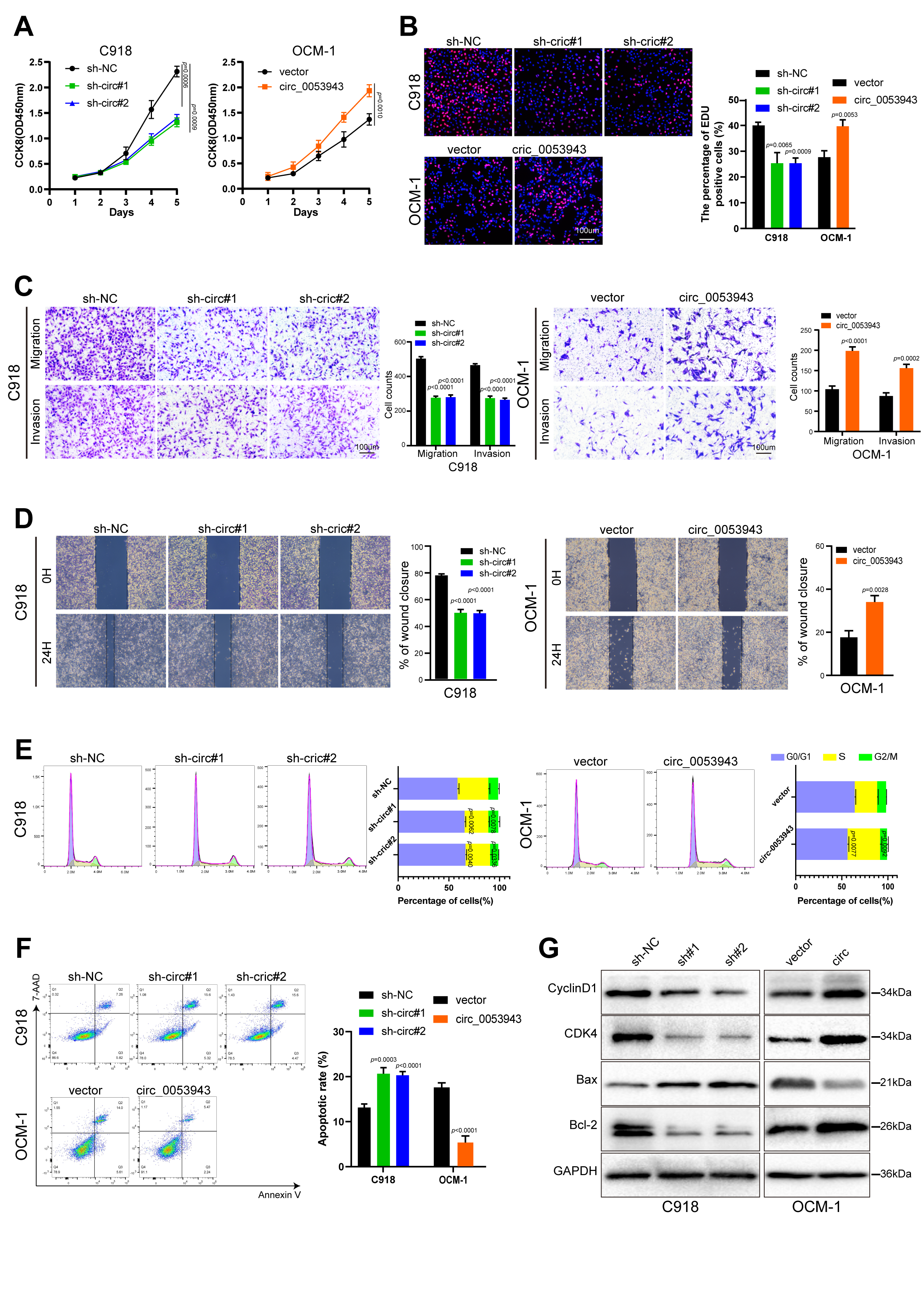

Supplement: FIGURE S2 [file OncolRes-32-45972-s002.tif]

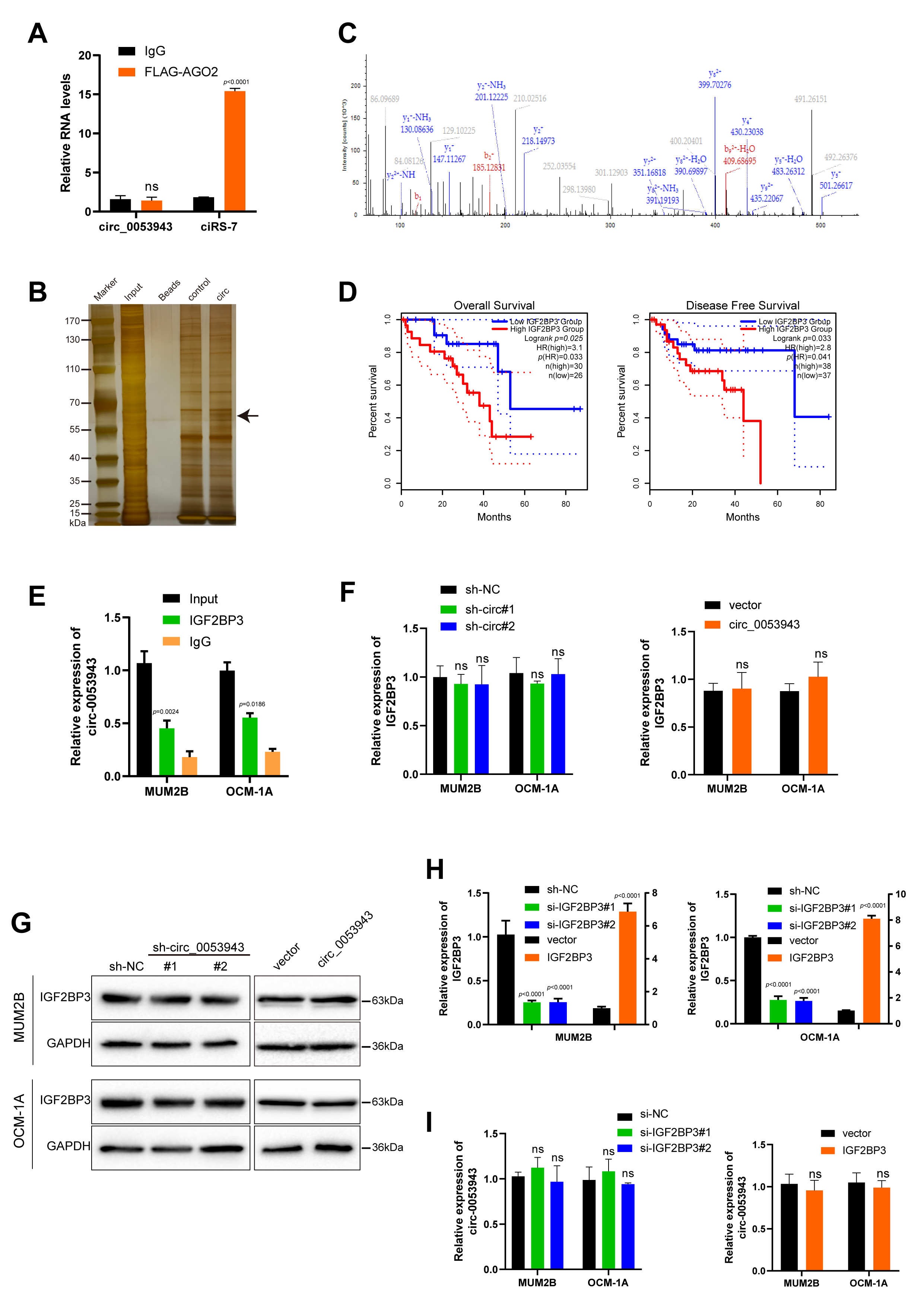

Supplement: FIGURE S3 [file OncolRes-32-45972-s003.tif]

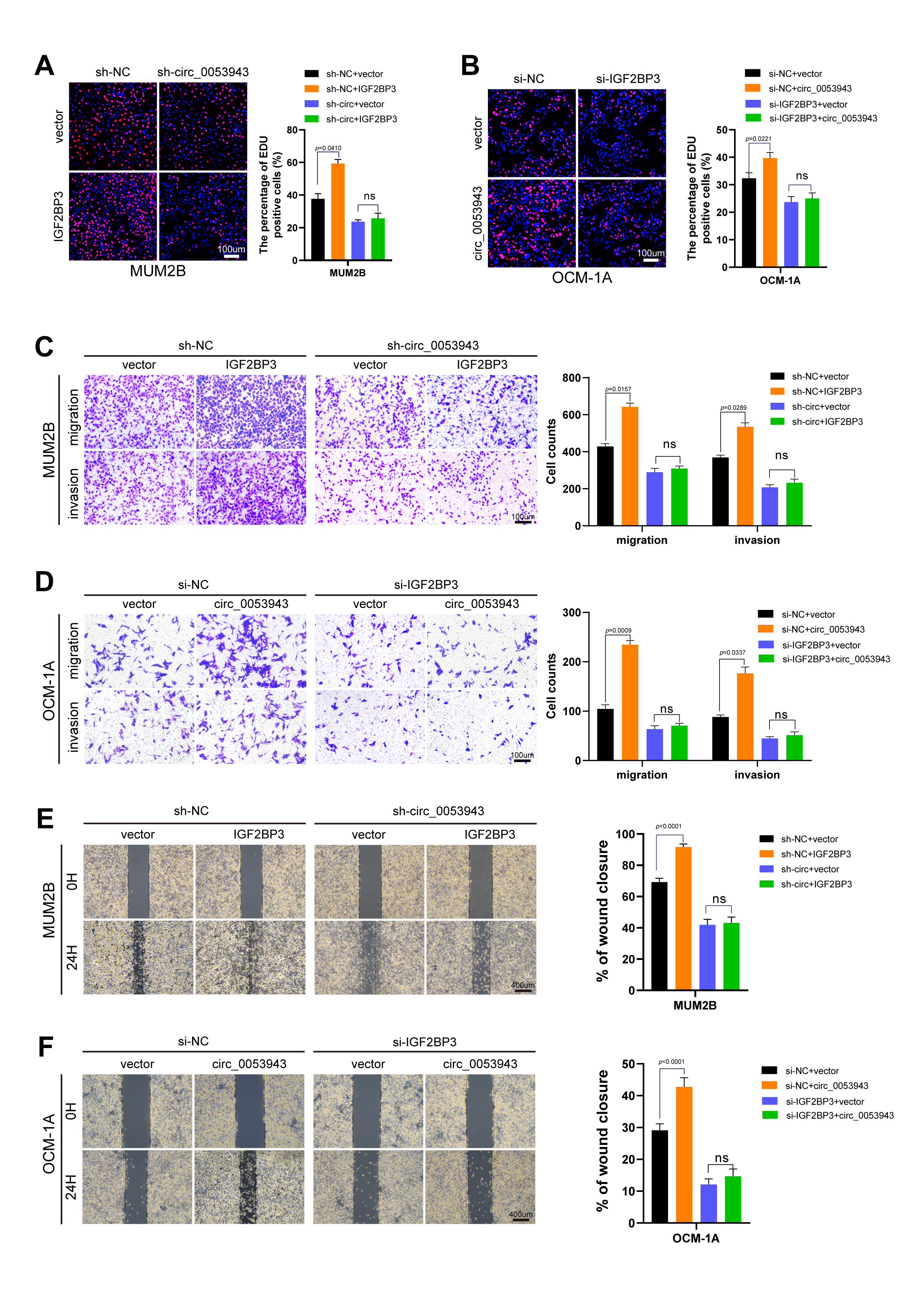

Supplement: FIGURE S4 [file OncolRes-32-45972-s004.tif]

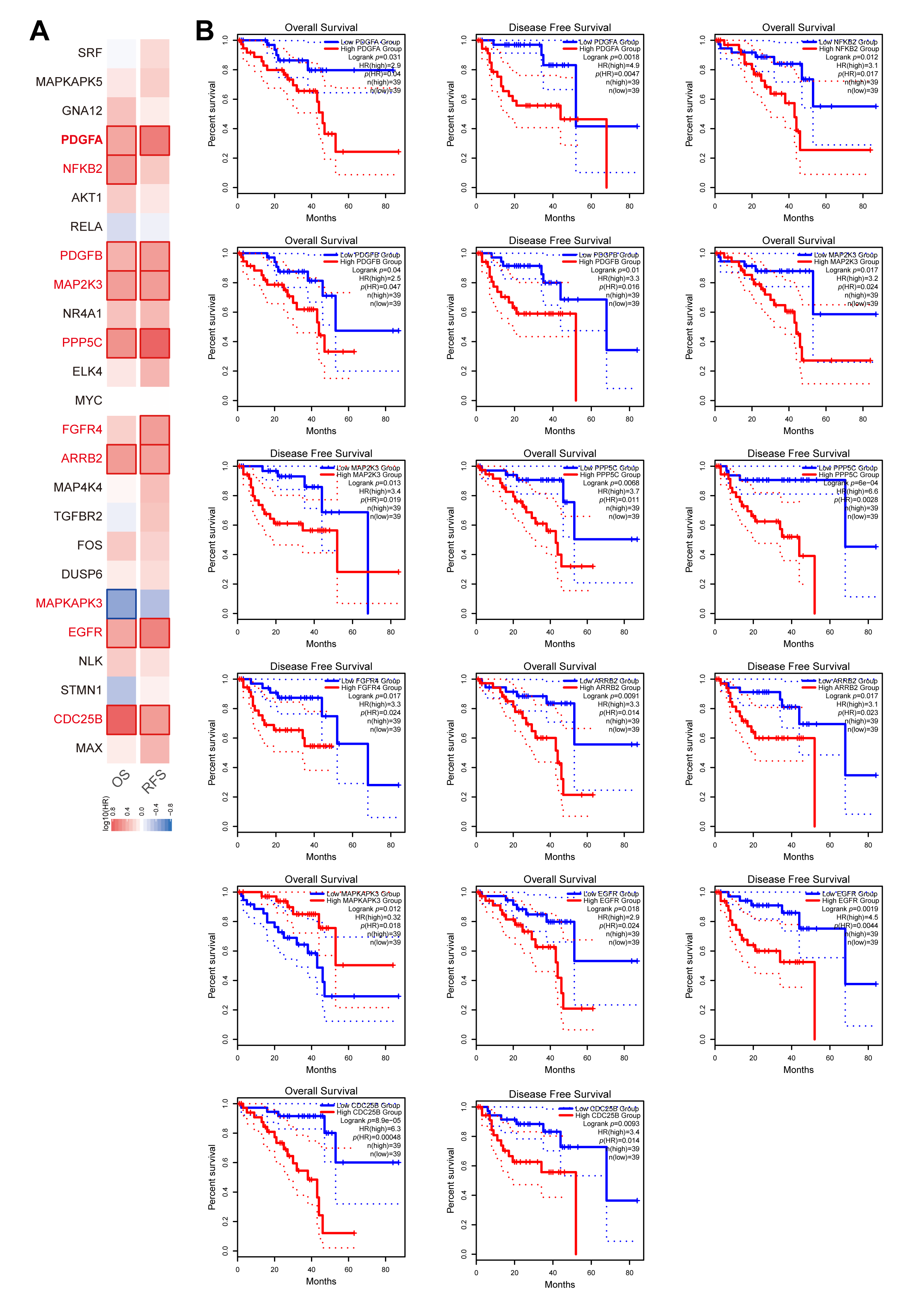

Supplement: FIGURE S5 [file OncolRes-32-45972-s005.tif]

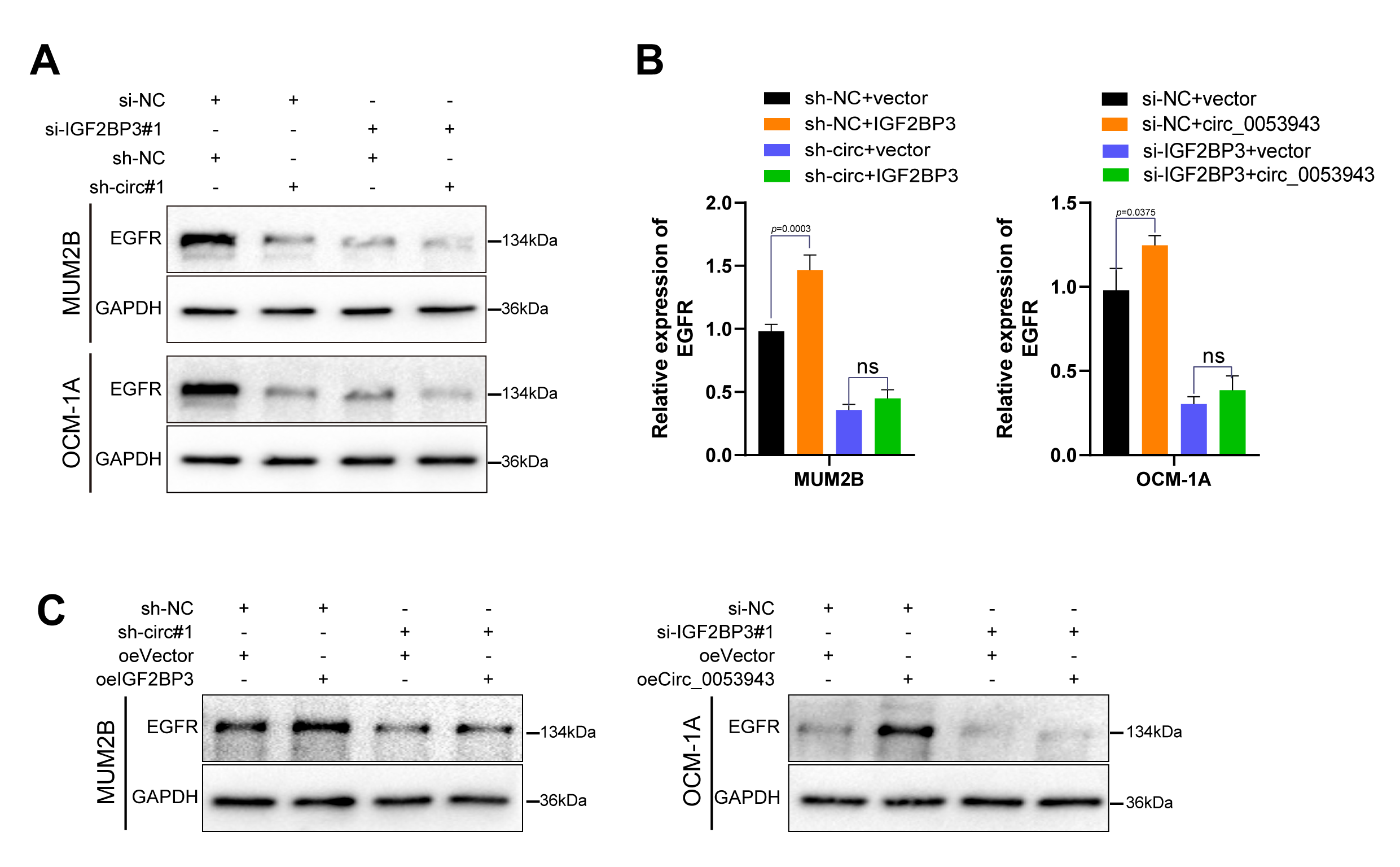

Supplement: FIGURE S6 [file OncolRes-32-45972-s006.tif]

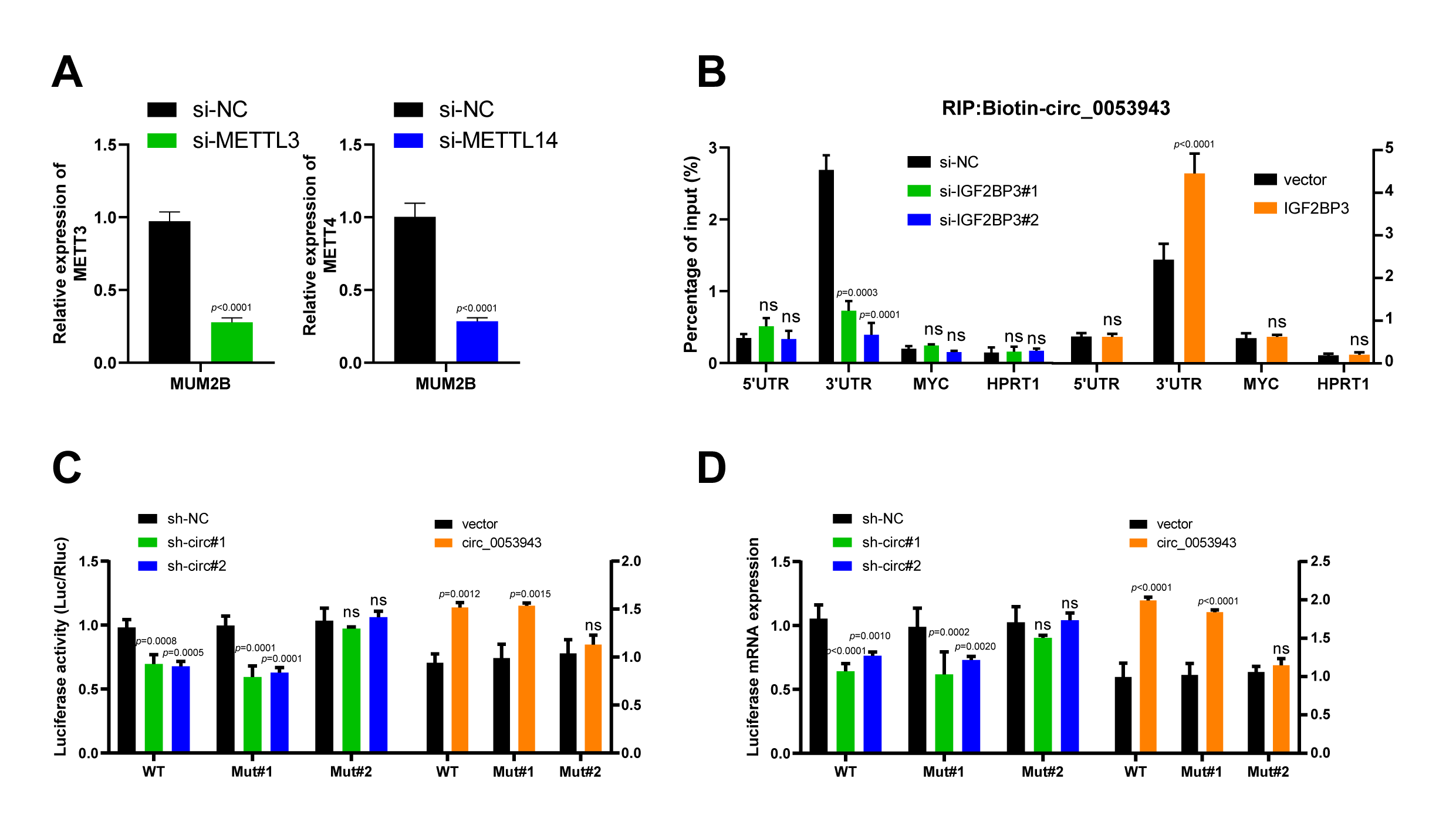

Supplement: FIGURE S7 [file OncolRes-32-45972-s007.tif]

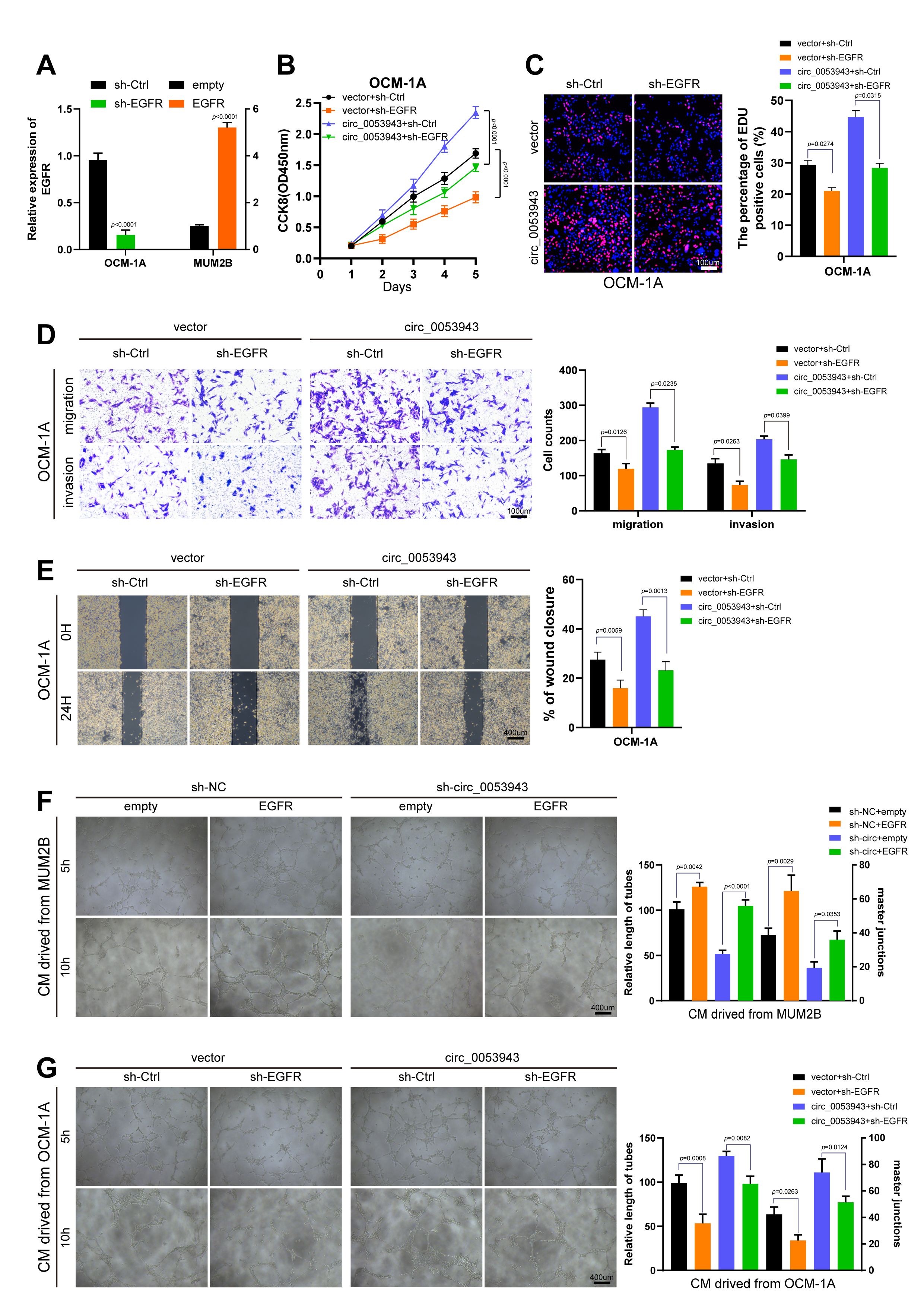

Supplement: FIGURE S8 [file OncolRes-32-45972-s008.tif]
