# Supplementary material for: Circ_0053943 complexed with IGF2BP3 drives uveal melanoma progression via regulating N6-methyladenosine modification of Epidermal growth factor receptor
Source: Oncol Res. 2024 Apr 23;32(5):983–98. doi: 10.32604/or.2024.045972 (PMC11055987; doi:10.32604/or.2024.045972)
Supplement: Supplementary file 9 [file OncolRes-32-45972-s001.docx]

**Supplementary Tables**

**Supplementary Table 1. The clinical characteristics of uveal melanoma patients.**

| **Patient No.** | **Sex (Female=0, Male=1)** | **Age** | **Stage**  **(AJCC 7th)** | **Thickn (mm)** |
| --- | --- | --- | --- | --- |
| 1 | 1 | 48 | T1 | 4 |
| 2 | 1 | 50 | T2 | 9 |
| 3 | 1 | 36 | T1 | 4 |
| 4 | 1 | 46 | T2 | 7 |
| 5 | 0 | 55 | T1 | 3 |

| **Supplementary Table 2. Primer sequences used in the study.** | | |
| --- | --- | --- |
| **Primer name** | | **Primer sequences** |
| hsa_circ_0053943 | Forward Primer | TTGTGAAAATGCACCAGCTC |
|  | Reverse Primer | AGCAATCGATCTTTCCAAGG |
| hsa_circ_0119872 | Forward Primer | CCATGGGATCAAGTGGCCTT |
|  | Reverse Primer | TCATTGGGAAATGCATGGAAGA |
| hsa_circ_0097065 | Forward Primer | ACAGCGGGCAGTATTAATGAAG |
|  | Reverse Primer | ACAAGTTGCTCATAATGGAAGGA |
| hsa_circ_0108830 | Forward Primer | TCACTTCTTGATGACTCTCAGG |
|  | Reverse Primer | GGCCAGAGTCCTCATCATGA |
| hsa_circ_0119873 | Forward Primer | CAAACTGTCACTGGTGCTGG |
|  | Reverse Primer | TTATTCTCCAGGCAGCCATG |
| hsa_circ_0119871 | Forward Primer | CACCCCTCTGATGACCCATT |
|  | Reverse Primer | GCTGTCATCACCTCCATTGC |
| hsa_circ_0056902 | Forward Primer | TGGATTTTTGACTTTGTAATGGA |
|  | Reverse Primer | CCTTGAGCAGCATGAAGACA |
| hsa_circ_0119874 | Forward Primer | ATCTCAAGCCAACCTTCTGC |
|  | Reverse Primer | GATGACTTCTGCCCTTTGCT |
| hsa_circ_0053943 convergent primers | Forward Primer | GTCTCCTCCAACGGCAATTA |
|  | Reverse Primer | GTAACGGAGAGCTGGTGCAT |
| RasGRP3 | Forward Primer | ACAGACTGCTTCTTCCATGC |
|  | Reverse Primer | GCAAGTCAGCGATGTTAGTGC |
| GAPDH | Forward Primer | TCAAGGCTGAGAACGGGAAG |
|  | Reverse Primer | TGATGATCTTGAGGCTGTTGTC |
| GAPDH convergent primers | Forward Primer | TGTACCATCAATAAAGTACCCTGTG |
|  | Reverse Primer | AAATCCGTTGACTCCGACCT |
| IGF2BP3 | Forward Primer | TCGAGGCGCTTTCAGGTAAA |
|  | Reverse Primer | AAACTATCCAGCACCTCCCAC |
| 18S | Forward Primer | ACACGGACAGGATTGACAGA |
|  | Reverse Primer | GGACATCTAAGGGCATCACA |
| U6 | Forward Primer | CTCGCTTCGGCAGCACA |
|  | Reverse Primer | AACGCTTCACGAATTTGCGT |
| EGFR | Forward Primer | GAGCCTCTGGATGGTGCAAT |
|  | Reverse Primer | GAGCCTCTGGATGGTGCAAT |
| ARRB2 | Forward Primer | TCCATGCTCCGTCACACTG |
|  | Reverse Primer | ACAGAAGGCTCGAATCTCAAAG |
| CDC25B | Forward Primer | ACGCACCTATCCCTGTCTC |
|  | Reverse Primer | CTGGAAGCGTCTGATGGCAA |
| FGFR4 | Forward Primer | CCATAGGGACCCCTCGAATAG |
|  | Reverse Primer | CAGCGGAACTTGACGGTGT |
| MAP2K3 | Forward Primer | GACTCCCGGACCTTCATCAC |
|  | Reverse Primer | GGCCCAGTTCTGAGATGGT |
| MAPKAPK3 | Forward Primer | AGATAATGCGGGATATTGGCAC |
|  | Reverse Primer | TGTGTAGAGTAGGTTTTCAGGCT |
| NFKB2 | Forward Primer | ATGGAGAGTTGCTACAACCCA |
|  | Reverse Primer | CTGTTCCACGATCACCAGGTA |
| PDGFA | Forward Primer | GCAAGACCAGGACGGTCATTT |
|  | Reverse Primer | GGCACTTGACACTGCTCGT |
| PDGFB | Forward Primer | CTCGATCCGCTCCTTTGATGA |
|  | Reverse Primer | CGTTGGTGCGGTCTATGAG |
| PPP5C | Forward Primer | CTGCGAGACTACGAGACGG |
|  | Reverse Primer | TCCGCTGTACTCATCCTCAAT |
| EGFR 3' UTR | Forward Primer | CCAGTATTGATCGGGAGAGC |
|  | Reverse Primer | CTGCGTGAGCTTGTTACTCG |
| EGFR 5' UTR | Forward Primer | TCCTTTGGGGCATAGATCAG |
|  | Reverse Primer | GCTGACCTGGAGGGAACATA |
| c-MYC CRD | Forward Primer | GCATACATCCTGTCCGTCCA |
|  | Reverse Primer | GTCGTTTCCGCAACAAGTCC |
| HPRT1 | Forward Primer | TGACACTGGCAAAACAATGCA |
|  | Reverse Primer | GGTCCTTTTCACCAGCAAGCT |

| **Supplementary Table 3. Knockdown shRNA or siRNA sequences used in this study.** | | |
| --- | --- | --- |
| **Targets** |  | **Sequences** |
| sh-circ_0053943#1 | shRNA-Forward (5'-3') | CCGGATCAACCTGCTCACGTTCACTCGAGATCAACCTGCTCACGTTCATTTTT |
|  | shRNA-Reverse (5'-3') | AATTCAAAAAATCAACCTGCTCACGTTCACTCGAGATCAACCTGCTCACGTTCA |
| sh-circ_0053943#2 | shRNA-Forward (5'-3') | CCGGCTGCTCACGTTCACTGATTCTCGAGCTGCTCACGTTCACTGATTTTTTT |
|  | shRNA-Reverse (5'-3') | AATTCAAAAACTGCTCACGTTCACTGATTCTCGAG CTGCTCACGTTCACTGATT |
| sh-circ_0053943#3 | shRNA-Forward (5'-3') | CCGGCACGTTCACTGATTACCAACTCGAGCACGTTCACTGATTACCAATTTTT |
|  | shRNA-Reverse (5'-3') | AATTCAAAAACACGTTCACTGATTACCAACTCGAGCACGTTCACTGATTACCAA |
| si-IGF2BP3#1 | shRNA-Forward (5'-3') | GATCCGCAAAGGATTCGGAAACTTCATTCAAGAGATGAAGTTTCCGAATCCTTTGCTTTTTTG |
|  | shRNA-Reverse (5'-3') | AATTCAAAAAAGCAAAGGATTCGGAAACTTCATCTCTTGAATGAAGTTTCCGAATCCTTTGCG |
| si-IGF2BP3#2 | shRNA-Forward (5'-3') | GATCCGCTGAGAAGTCGATTACTATCTTCAAGAGAGATAGTAATCGACTTCTCAGCTTTTTTG |
|  | shRNA-Reverse (5'-3') | AATTCAAAAAAGCTGAGAAGTCGATTACTATCTCTCTTGAAGATAGTAATCGACTTCTCAGCG |
| si-METTL3 | shRNA-Forward (5'-3') | GATCCGGATACCTGCAAGTATGTTCATTCAAGAGATGAACATACTTGCAGGTATCCTTTTTTG |
|  | shRNA-Reverse (5'-3') | AATTCAAAAAAGGATACCTGCAAGTATGTTCATCTCTTGAATGAACATACTTGCAGGTATCCG |
| si-METTL14 | shRNA-Forward (5'-3') | GATCCGCTGGACTTGGGATGATATTATTCAAGAGATAATATCATCCCAAGTCCAGCTTTTTTG |
|  | shRNA-Reverse (5'-3') | AATTCAAAAAAGCTGGACTTGGGATGATATTATCTCTTGAATAATATCATCCCAAGTCCAGCG |
| sh-EGFR | shRNA-Forward (5'-3') | CCGGGCCACAAAGCAGTGAATTTATCTCGAGATAAATTCACTGCTTTGTGGCTTTTTG |
|  | shRNA-Reverse (5'-3') | CCGGGCCACAAAGCAGTGAATTTATCTCGAGATAAATTCACTGCTTTGTGGCTTTTTG |

| **Supplementary Table 4. List of primary antibodies.** | | | |
| --- | --- | --- | --- |
| **Name** | **Catalog** | **Company** | **Application** |
| Anti-Bax antibody | ab32503 | Abcam | WB |
| Anti-Bcl-2 antibody | ab32124 | Abcam | WB |
| Anti-CDK4 antibody | ab108357 | Abcam | WB |
| Anti-CyclinD1 antibody | ab134175 | Abcam | WB |
| Anti-GAPDH antibody | #60004-1-Ig | Proteintech | WB |
| Anti-IGF2BP3 antibody | ab177477 | Abcam | WB, IF, IP |
| Anti-FARSB antibody | ab126950 | Abcam | WB |
| Anti-KPNA2 antibody | ab170495 | Abcam | WB |
| Anti-FLAG antibody | ab205606 | Abcam | WB, IP |
| Anti-ERK antibody | ab50011 | Abcam | WB |
| Anti-p-ERK antibody | ab184699 | Abcam | WB |
| Anti-JNK antibody | 9252S | CST | WB |
| Anti-p-JNK antibody | 9251S | CST | WB |
| Anti-p38 antibody | 8690S | CST | WB |
| Anti-m^6^A antibody | ab52894 | Abcam | WB, IHC |
| Anti-p-p39 antibody | #202003 | Synaptic Systems | IP |
| Anti-EGFR antibody | 4511S | CST | WB |
| Anti-Ki67 antibody | 27309-1-AP | Proteintech | IHC |

| **Supplementary Table 5. The sequence of probes target circ_0053943 for RNA pulldown assay.** | |
| --- | --- |
| **Pull Down probe sequence** | |
| hsa_circ_0053943(3bio)_Probe | ATCAGTGAACGTGAGCAGGT |
| hsa_circ_0053943(3bio)_scramble | GTCGAGAATTGACACGAGTG |

**Supplementary Table 6. Differential proteins of circ_0053943 specifical probe in MUM2B.**

| **ID** | | | | | |
| --- | --- | --- | --- | --- | --- |
| ACTG2 | CCT2 | UBQLN2 | EIF4H | EIF3L | DXO |
| NME1 | PSMC2 | TXNRD1 | PSMG1 | EMC2 | TCERG1 |
| YWHAQ | PSMC1 | NUDC | CANX | DPM3 | SNX3 |
| EIF4A2 | TUFM | SET | POLR2H | EDF1 | SERPINC1 |
| SFN | GNB4 | MCM6 | SLC7A5 | HNRNPA3 | PPP1CA |
| CLIC4 | NAP1L1 | RPS21 | CSTB | PODXL | CIRBP |
| CAP1 | AHSG | SBDS | TOR1AIP1 | ZNF826P | CSDE1 |
| PDIA6 | APRT | MARCKS | CORO1C | NAA15 | ITGB1 |
| HNRNPA1 | UCHL1 | HADHA | DYNLRB2 | PRDX4 | AK2 |
| BZW1 | APOBEC3C | EIF3I | PSME3 | TOMM20 | GLS |
| HSPA4 | PLS3 | ITGA3 | PSMC6 | ARPC5 | SLC1A5 |
| CALR | DNAJA1 | RTCB | PSMD11 | KPNA2 | ATP1B3 |
| TRAP1 | S100A13 | G3BP2 | FARSB | CTNND1 | AKR1B1 |
| TPM3 | SSB | PCNA | RANBP1 | FKBP4 | AKAP12 |
| CDC37 | RAB5C | CALM3 | PSMA5 | RPL13A | ACTR1A |
| RBMS2 | RPL14 | KHDRBS1 | RPL21 | PGLS | SHMT2 |
| DYNC1H1 | RPA2 | TMX1 | S100A16 | ATP6V1A | EIF3E |
| HNRNPAB | DYNLL2 | PSMC3 | CSRP1 | PSMC4 | NANS |
| PRMT1 | S100A10 | PFN2 | LAMP1 | PGM2 | KLC1 |
| PNP | MT1X | RPLP1 | MME | PGM1 | SSR4 |
| PPP2CB | PSMD13 | SORD | BTF3L4 | RCC2 | AP2B1 |
| IPO5 | IDH3B | COX6C | DDB1 | OCIAD1 | SRPRB |
| CNBP | SLC9A3R1 | UCHL3 | BCAP31 | PFKP | ARHGDIA |
| RPL7A | PDLIM5 | G6PD | SNRPA | GMPS | ATP12A |
| USP5 | GLO1 | EEF1E1 | IGF2BP3 | PRPF3 | VPS35 |
| TOMM7 | PSMA4 | PFAS | TRIM4 | DDX39A | EIF3F |
| TMEM258 | PDCD10 | OXA1L | PDCD5 | EIF3H | LRPPRC |
| DHX9 | TCOF1 | PPA1 | SNRNP70 | NFIB |  |

**Supplementary Table 7. RNA-binding protein (RBPs).**

| **ID** | | | | | | | |
| --- | --- | --- | --- | --- | --- | --- | --- |
| TARDBP | SPTAN1 | EEF1A1 | ANP32B | RPL11 | XRCC6 | PRPF40B | PPP4R2 |
| GRN | MKI67 | DDX39B | PPP1R8 | ADD1 | GANAB | PRPF40A | RPF1 |
| LRP1 | UBC | ELAVL1 | MRPL10 | HUWE1 | SYNE1 | RRS1 | UTP18 |
| HSD17B10 | YWHAE | RPS3A | NOC3L | PSMD4 | TCERG1 | MTDH | ENOX1 |
| VCP | CTNNA1 | ADARB1 | RRP1 | HDAC2 | ACAA2 | HLTF | TWISTNB |
| FUS | APEX1 | C4BPA | EIF4A1 | SUMO1 | ROCK2 | EXOSC9 | RBMY1J |
| JUN | RAN | PPP1R9B | ZRSR2 | TUFM | PURA | CHD3 | TWF2 |
| EIF2AK2 | SNRNP70 | PSMC1 | SREK1 | PCBP2 | POLR2A | SCNM1 | SKIV2L2 |
| ATXN2 | HSPA1A | PEG10 | PRPF18 | NKRF | RPS15 | RBM5 | DHX8 |
| NQO1 | TXN | C1QBP | ZNF326 | RPS8 | RPL7A | NOP14 | CDK12 |
| RPS27A | ELAC2 | UBE2I | RRP12 | RPS9 | RPS20 | UBTF | CPSF1 |
| HNRNPA1 | RBM25 | NACA | ZCCHC24 | RBM3 | PUF60 | SNW1 | CDC40 |
| AATF | ALDH18A1 | HMGCS2 | ADARB2 | ANXA2 | HSPA1L | ZCCHC6 | CCDC94 |
| EIF2S1 | YWHAH | NCL | TRA2B | PSPC1 | RPS2 | AKAP8L | IMP4 |
| DCN | CAST | GTPBP4 | UTP20 | SF3B3 | RPS3 | ZFP36 | DDX47 |
| CDKN2A | GRB2 | MARK2 | CSTF2T | SLC25A5 | WBSCR22 | LUC7L2 | PCBP4 |
| HSPD1 | TPT1 | SORBS2 | CPEB3 | PPIL4 | RPS27 | SRSF4 | CPNE3 |
| HNRNPA2B1 | YLPM1 | NOLC1 | RBM45 | SYNCRIP | RPL23A | SUMO2 | CCDC12 |
| RBM8A | EBNA1BP2 | BICC1 | HLA-A | RBM19 | RPS16 | EIF3E | STAU1 |
| TFAM | TTF2 | YWHAZ | TARDBP | C14orf166 | MANF | ECH1 | RBM34 |
| WT1 | MTPAP | ELAVL2 | PFN1 | EEF1G | NUCKS1 | RBMXL1 | CCDC137 |
| CSTB | UBE2D1 | PPP1R10 | MATR3 | RBMS2 | RPL13 | LUC7L | CGGBP1 |
| CALR | TCP1 | ELAVL3 | HSPB1 | RBMS1 | LSM14A | BTF3 | BCAS2 |
| HSPA8 | ATP5C1 | CKAP4 | SMN1 | PDIA3 | RPL28 | PABPC4 | ZC3H18 |
| FLNA | FASN | PPP2CA | GFM1 | RAVER1 | YWHAG | PPIL1 | NOL8 |
| PPARGC1A | UBE2L3 | MSI1 | NAP1L1 | GOT2 | RPL10A | SAFB | HEATR1 |
| DYNC1H1 | GPX4 | HNRNPH3 | TNPO1 | HSP90AB1 | RPS11 | ZFC3H1 | NSA2 |
| MECP2 | PRKRA | EXOSC1 | NOP56 | FAM98A | U2AF1 | YTHDF3 | NOA1 |
| HMGB1 | HNRNPDL | BMS1 | DUSP11 | HNRNPD | TBCA | ZCCHC17 | RRAGC |
| HSPG2 | RBM4 | HIST1H1B | SSR1 | HNRNPU | TRAP1 | UTP14A | SNRPA1 |
| TFRC | DDX21 | RPS23 | PRMT1 | HNRNPL | PPHLN1 | PHF5A | EIF1AX |
| HSPA9 | MAZ | RPL4 | UPF1 | HNRNPA3 | USP36 | TOP1 | BRIX1 |
| ADAR | PRDX1 | CCAR1 | XRN2 | SERBP1 | HIST1H1C | MYOD1 | NOL6 |
| RTN4 | QKI | ANXA7 | RBFOX1 | SF3A3 | NMT1 | BST2 | NIP7 |
| ATP5A1 | NDUFV3 | CDK11B | PABPN1 | RPL19 | SRRM2 | PPIG | MBNL3 |
| STXBP1 | RPS6 | TFB1M | SERPINH1 | SF3A1 | SMG1 | FBL | SLTM |
| TPI1 | ANXA11 | EIF3A | UBA1 | RPS14 | MRPS9 | EXOSC10 | TCEA1 |
| LBR | SCG3 | ATXN2L | TAF15 | RPS5 | ZMAT5 | TBL2 | MRPS26 |
| NME1 | ADK | PRMT5 | FMR1 | ERAL1 | WBP11 | IFI16 | KRI1 |
| HSP90AA1 | SNRPA | SEC23IP | SON | RPL14 | NOVA1 | RPS17 | MACF1 |
| FLNB | RSRC1 | IFIT5 | NPM1 | SNRPD2 | UBE2N | SBDS | FNDC3B |
| HSPA5 | TSNAX | SUPV3L1 | ZNFX1 | RPL12 | U2AF2 | METAP2 | MAGOH |
| HADHB | IMMT | RPP30 | AQR | SSBP1 | WDR75 | RPL7 | PATL1 |
| ASS1 | ARF1 | TIAL1 | DBR1 | NUDT21 | PRPF8 | CISD2 | SNRNP27 |
| ACTB | FARSA | PPRC1 | PPIA | UBAP2L | CLTC | EZR | UTP6 |
| ALDOA | EIF4E | RBM17 | SF3B4 | UBFD1 | POLR2B | RPL5 | THOC1 |
| ENO1 | CELF2 | GDI2 | LGALS3 | UBAP2 | CHERP | PRKDC | SRSF8 |
| ACTN4 | CELF1 | HNRNPF | SF1 | NGRN | POLR2I | XRCC5 | RBMY1D |
| EEF2 | PEBP1 | SCAF11 | HNRNPK | CCT3 | POLR2J | RDX | TNRC6B |
| FDPS | POLR2L | TFB2M | PPP2R1A | CCT6A | POLR2C | TIA1 | TDRD3 |
| PARP1 | ABT1 | ZYX | KRT18 | NONO | POLR2D | HMGN2 | XRN1 |
| DNM1 | ELAVL4 | ANP32A | LGALS1 | CCT4 | HSPE1 | RIMS1 | PCDH20 |
| PCSK9 | KPNA2 | KHSRP | KPNB1 | TXNL4B | POLR2F | CORO1A | PTRH1 |
| FKBP4 | SMNDC1 | GLRX3 | PABPC1 | CPSF6 | POLR2K | ZFP36L1 | ZC3H7A |
| PTPN1 | HSP90B1 | SRSF3 | EWSR1 | DHX15 | POLR2E | POU5F1 | TRPT1 |
| SFPQ | HSPA1B | PRRC2C | CDC5L | DDX5 | POLR2G | HELZ | LARP4 |
| RP9 | PTBP1 | PPIL2 | HNRNPH1 | DDX42 | POLR2H | MYH9 | TNRC6A |
| P4HB | ACTN1 | BZW2 | DDX1 | GIGYF2 | LIN28A | HNRNPC | ESF1 |
| CS | MDH2 | MRPL43 | PNPT1 | EIF4G1 | NCBP1 | BAG4 | CPSF4 |
| TUBB | MAPRE1 | IFIT2 | HARS2 | DDX41 | CLK1 | USO1 | MRPS5 |
| RNPC3 | DSP | SRPK1 | FASTKD2 | HEATR6 | FAM120A | RPS21 | RBM33 |
| STAU2 | PRPF6 | EIF5A2 | LIN28B | RBM39 | RBFOX3 | RPL27 | TBL3 |
| RALY | PRPF4 | SRP72 | PES1 | LARP1 | RBFOX2 | LRRC59 | RBM6 |
| FSCN1 | EIF2S2 | PTBP2 | UPF3B | DHX38 | BRDT | CAPRIN1 | RPS27L |
| TOP2A | SNRPD3 | MRPL28 | MRPL44 | BCLAF1 | RBPMS | CLNS1A | HNRNPH2 |
| FIP1L1 | SNRPD1 | KHDRBS3 | EPPK1 | PPAN | FXR1 | DARS | MRPS11 |
| ZC3HAV1 | SNRPE | HMGB2 | MRPL3 | TPD52L2 | NOMO1 | EIF1B | MRPS14 |
| OASL | SNRPG | SND1 | SNIP1 | NOP2 | API5 | SF3B5 | MRPS27 |
| RNPS1 | GAR1 | SART1 | THOC6 | PPIE | RPL3 | RPS25 | DNAJC8 |
| BUD13 | SNRPF | KTN1 | NUFIP2 | ILF3 | TES | SYF2 | NAF1 |
| NCOA5 | SNRPB2 | PSIP1 | ZFR | SAMD4A | SUPT5H | EDF1 | DHX33 |
| HOXB6 | EIF4H | YTHDF1 | WDR43 | CWC22 | ACIN1 | GRSF1 | SMU1 |
| RRP9 | SRSF2 | H1F0 | TOP3B | SRP14 | MYO18A | SEC61A1 | NOL7 |
| XAB2 | NOVA2 | USP10 | RPL21 | YTHDC1 | CTNNBL1 | ARCN1 | MRPL20 |
| ZC3H10 | CRKL | ABCF1 | R3HDM2 | ZC3H4 | RPL30 | TBRG4 | SRP54 |
| NLRP11 | CSRP1 | RPL10 | G3BP1 | PRMT7 | C15orf52 | PAK1IP1 | SPATS2 |
| DHX16 | FXR2 | EIF4G3 | MOV10 | PCF11 | DDX17 | FASTKD5 | ANKHD1 |
| DROSHA | KHDRBS1 | HIST1H1E | SRSF10 | ALDH6A1 | DIAPH1 | ZNF593 | NAT10 |
| SNRNP35 | LSM1 | RPL26 | DDX3X | ALKBH5 | POP1 | RBBP6 | LARP4B |
| MRPL19 | LSM7 | RPL17 | CEBPZ | EIF5B | FUBP1 | TRIM71 | MRPL42 |
| AFF2 | TGS1 | SRPK2 | YBX1 | TCF20 | XPO5 | TXNL4A | ZCCHC9 |
| TSEN54 | LSM5 | BOP1 | MBNL2 | MRPS24 | BYSL | NBPF10 | CWC15 |
| RARS2 | LSM4 | BZW1 | RPSA | SAMD4B | SEC61B | DAZL | RBMY1A1 |
| DDX20 | LSM3 | SRRT | MYBBP1A | WDR36 | IVNS1ABP | LGTN | YTHDF2 |
| RPGR | LSM6 | CDK13 | ANKRD17 | MRPL9 | DHX32 | TCF25 | SAMSN1 |
| RBM10 | GEMIN8 | APEH | SRSF1 | TRUB2 | ESRP1 | CPEB1 | RRP1B |
| TSEN2 | HNRNPM | SECISBP2 | HIST1H1D | CPSF2 | JMJD6 | DAZ3 | GTF2E2 |
| CNBP | GEMIN5 | RBM15 | SUCLG1 | SSRP1 | EIF3C | PUM2 | MRPL54 |
| TSEN34 | S100A4 | SRSF6 | ZFP36L2 | ALYREF | EIF4B | APOBEC3B | LARP1B |
| HNRNPR | IGF2BP3 | PUS1 | XIRP1 | DEK | KIAA1324 | DAZ2 | DCAF13 |
| PLEC | SPEN | SARNP | NUSAP1 | AHNAK | EIF4A2 | SSBP3 | OTUD4 |
| YARS | SPTBN1 | ZCCHC7 | NOL3 | RSL1D1 | LYAR | ZNF622 | DDX52 |
| IBA57 | NAP1L4 | TUT1 | CHD2 | RBMX | GTF2F1 | UNK | HNRNPA1L2 |
| LAS1L | NHP2 | ZCCHC11 | RPL36A | HIST2H4B | RBM47 | RPS4X | BCCIP |
| PHF6 | SSB | PRRC2A | RPL24 | HIST1H4H | FASTKD3 | ZNF598 | RRP15 |
| TACO1 | HDGF | C1orf35 | RPL23 | SRSF5 | EIF3D | STRBP | EIF3G |
| DKC1 | ARHGEF1 | PRRC2B | RPL37A | SRSF9 | FASTK | RBPMS2 | KIAA1429 |
| PRPF31 | CANX | DUSP14 | PNN | A1CF | PKN2 | NKAP | SDAD1 |
| PQBP1 | RPL32 | SEC63 | MCAT | WDR46 | PCBP1 | SRPK3 | ASCC3 |
| MYO5A | COL14A1 | SRFBP1 | H1FX | RPS18 | L1TD1 | RC3H2 | POLDIP3 |
| SMC1A | FNDC3A | UTP3 | FAM46A | FBRSL1 | MRPL13 | SCAF4 | SECISBP2L |
| LRPPRC | PELP1 | PTCD2 | CLK3 | RPL6 | EIF3CL | RRBP1 | TRMT1 |
| GNL3 | EIF5A | DIS3 | CSNK1E | PRR3 | PRPF38B | CIR1 | ZCCHC8 |
| PARN | SLC3A2 | YARS2 | PPP1CC | HK2 | GTPBP1 | WTAP | CRNKL1 |
| SNRNP200 | FKBP3 | EFTUD2 | PUM1 | NUMA1 | RTF1 | SRRM4 | SF3B2 |
| ALG13 | IGF2BP2 | TPR | GOLGB1 | IGF2BP1 | NOC2L | LARP7 | FTSJ3 |
| KIF1C | HNRNPAB | RBM20 | CPEB4 | FAU | TAGLN2 | SAP18 | REXO4 |
| RNU4ATAC | TMSB4X | NSUN5 | CASC3 | CWC27 | CNOT1 | EIF4G2 | DNAJC21 |
| MTO1 | ILF2 | RPL35A | DHX9 | EIF3H | NOL10 | DDX10 | TRMT6 |
| SNRPB | TRIM28 | RPS26 | DDX18 | DDX39A | POLRMT | EIF2D | ZC3H11A |
| SNRPN | PPIB | RPS19 | DDX6 | NAA15 | TRMT1L | EIF4E2 | C1orf52 |
| CLP1 | XPO1 | NSUN2 | RPL31 | SF3B1 | RPS13 | PURB | SLIRP |
| SNTB2 | RPLP0 | RPS24 | LTA4H | KCTD12 | RPL18A | RPL22 | CSTF1 |
| DDX23 | PTRF | RPS7 | THOC2 | OLA1 | SRSF7 | NASP | ZCCHC3 |
| MBNL1 | STIP1 | RPL15 | ZC3H14 | ZMAT3 | AKAP17A | HTATSF1 | RBM46 |
| TNS1 | IMP3 | RPS10 | ZNF346 | ZAK | MEX3A | SRSF11 | DIMT1 |
| LSM2 | RPL29 | RPS28 | MRPL40 | PA2G4 | DIDO1 | HEXIM1 | DDX46 |
| RBM28 | EIF4A3 | SARS2 | DAZ4 | EED | ERH | CCDC47 | CPSF7 |
| PRPF3 | DCD | FRG1 | DAZ1 | RPS12 | RBM14 | SART3 | CDC42EP4 |
| TCOF1 | TRIP6 | TSFM | TRIM25 | SCAF1 | AKAP1 | G3BP2 | PNO1 |
| NXF1 | AKAP8 | PDCD11 | DHX57 | EIF3J | HMG20B | RBMXL2 | CCDC124 |
| EIF4ENIF1 | PWP1 | PLRG1 | DDX28 | WDR33 | HSPA7 | PUS7 | CIRBP |
| DUT | NPM3 | MPHOSPH10 | DDX27 | CSTF2 | BUD31 | FAM133B | DNAJC17 |
| DYNC1LI1 | SLU7 | PDAP1 | DNTTIP2 | MRPS30 | MRPS23 | RRP7A | RBM12B |
| SLBP | MEX3C | GRWD1 | PPAN-P2RY11 | MRPL41 | ARL6IP4 | CHCHD1 | SPATS2L |
| MKRN1 | POLR1E | RPF2 | HMGN5 | DUS3L | WDR25 | SLC16A3 | UBE2O |
| MAP4 | DZIP3 | PWP2 | CD3EAP | METTL16 | PPIH | R3HDM1 | RPL13A |
| TFIP11 | MEPCE | FCF1 | DHX35 | ZRSR1 | S100A16 | MRPL45 | TRMT2A |
| NAA38 | USP39 | SRP68 | PAPOLA | RPS19BP1 | THOC3 | ERI3 | RBMS3 |
| SUB1 | WDR3 | SUPT16H | WDR6 | RBM7 | NTPCR | C1orf131 | TRA2A |
| MFAP1 | CCDC86 | ZNHIT6 | PNISR | MRPS31 | PRPF4B | C1orf204 | RBMY1F |
| DNAJC2 | DDX55 | NOL11 | MRPS28 | SURF2 | RPL8 | MTIF2 | PURG |
| SUPT6H | DHX29 | NOC4L | GPATCH8 | ZNF385A | CPSF3 | MTHFSD | KRR1 |
| PRPF19 | DDX51 | TEX10 | PARP12 | REPIN1 | LRRC47 | FAM50A | YTHDC2 |
| THOC5 | DDX54 | NOP16 | RPN1 | MRPL32 | WBP4 | FAM32A | EXOSC2 |
| DHX30 | APOBEC3F | RRP36 | C11orf68 | ZC3H7B | SUGP2 | MRPS15 | NOMO2 |
| YBX2 | TRIM56 | MAK16 | NOM1 | RBMX2 | THOC7 | NOL12 | CWC25 |
| HNRNPLL | FAM103A1 | FARSB | RPL36 | LSM14B | CLK4 | CELF3 | CCDC9 |
| KHDRBS2 | NUPL2 | MRTO4 | MKRN2 | NMD3 | RPL35 | MAGOHB | GSPT2 |
| AEN | APOBEC3H | EMG1 | DDX50 | MRPL15 | PTCD3 | MRPL39 | NGDN |
| LUC7L3 | IPO5 | HNRNPCL1 | DAZAP1 | RPUSD3 | STRAP | PABPC5 | RBM38 |
| C7orf50 | RRP8 | GNL2 | EIF5 | RPUSD4 | ZC3H13 | CPEB2 | NVL |
| PDIA4 | RC3H1 | HNRNPUL1 | DAP3 | PHAX | ZC3H8 | MRPL1 | NOMO3 |
| RBM26 | HMGB3 | WDR77 | C11orf31 | MRPL11 | CD2BP2 | CELF5 | DIEXF |
| SURF6 | DHX36 | GLTSCR2 | ZNF768 | MRPL4 | THRAP3 | CELF4 | FLYWCH2 |
| RBM23 | DGCR14 | GSPT1 | HDLBP | C12orf43 | CLK2 | FAM120C | CSDE1 |
| LSM10 | HERC5 | ZNF579 | NOP58 | LSG1 | SLC25A11 | RPP25 | RPL27A |
| SRRM1 | URB2 | ZNF638 | DDX31 | MRPL14 | ZRANB2 | CELF6 | MEX3B |
| RPS15A | DDX56 | PRPF38A | GPATCH4 | MRPS21 | U2SURP | ISG20L2 | UTP15 |
| FUBP3 | WDR83 | EXOSC4 | CSTF3 | SRP19 | RBM15B | MRPS12 | MRPS7 |
| ZC3H15 | APOBEC3C | SNRPC | HNRNPUL2 | MRPL27 | RBM27 | PEF1 | ESRP2 |
| HNRNPA0 | SAFB2 | EXOSC6 | UCHL5 | GEMIN7 | THUMPD1 | RBM12 | MYEF2 |
| PDIA6 | DMGDH | TSR1 | CCDC59 | NOSIP | RBMY1B | CNOT4 | DDX24 |
| RCC2 | MSI2 | URB1 | INTS6 | ETF1 | RBMY1E | FYTTD1 |  |

| **Supplementary Table 8. The public database mentioned in the article.** | |
| --- | --- |
| **Name** | **website address** |
| SRAMPA | http://www.cuilab.cn/sramp |
| RMBase V2.0 | http://rna.sysu.edu.cn/rmbase/index.php |
